# Supplementary material for: Identification of c-di-GMP/FleQ-Regulated New Target Genes, Including cyaA, Encoding Adenylate Cyclase, in Pseudomonas putida
Source: mSystems. 2021 May 11;6(3):e00295-21. doi: 10.1128/mSystems.00295-21 (PMC8125075; doi:10.1128/mSystems.00295-21)
Supplement: TABLE S1 [file mSystems.00295-21-st001.doc]

| Gene_id | FoldChange | *P* val | Genename | Description |
| --- | --- | --- | --- | --- |
| PP_0089 | 2.081 | 3.12E-05 | *osmC* | stress-induced peroxiredoxin |
| PP_0115 | 2.244 | 1.00E-09 | *katE* | hydroperoxidase |
| PP_0137 | 6.284 | 6.10E-85 | *gltP* | glutamate/aspartate-proton DAACS transporter |
| PP_0168 | 2.66 | 3.12E-19 | *lapA* | putative surface adhesion protein |
| PP_0321 | 3.821 | 4.79E-64 | *ltaE* | low specificity l-threonine aldolase |
| PP_0323 | 2.013 | 1.22E-07 | *soxB* | sarcosine oxidase subunit beta |
| PP_0355 | 2.631 | 0.00049138 | *-* | two-component system response regulator |
| PP_0490 | 2.062 | 8.82E-21 | *fdoH* | formate dehydrogenase-O subunit beta |
| PP_0596 | 5.222 | 2.02E-246 | *-* | omega-amino acid--pyruvate aminotransferase |
| PP_0597 | 4.039 | 3.81E-66 | *mmsA-I* | methylmalonate-semialdehyde dehydrogenase |
| PP_0620 | 3.801 | 1.37E-06 | *-* | GntR family transcriptional regulator |
| PP_0658 | 2.395 | 3.50E-08 | *-* | homocysteine S-methyltransferase family protein |
| PP_0702 | 3.472 | 0.0020299 | *-* | MFS transporter |
| PP_0708 | 2.699 | 7.81E-07 | *-* | betaine-aldehyde dehydrogenase |
| PP_0793 | 2.032 | 2.90E-18 | *fruB* | PTS fructose transporter subunit EI/HPr/IIA |
| PP_0794 | 4.189 | 1.46E-08 | *fruK* | 1-phosphofructokinase monomer |
| PP_0795 | 2.608 | 2.09E-28 | *fruA* | PTS fructose transporter subunit IIBC |
| PP_1015 | 2.009 | 3.63E-09 | *gtsA* | mannose/glucose ABC transporter substrate-binding protein |
| PP_1018 | 2.689 | 2.46E-05 | *gtsD* | mannose/glucose ABC transporter ATP binding protein |
| PP_1039 | 24.200 | 0.0017221 | *tatC-I* | Sec-independent protein translocase protein |
| PP_1059 | 3.626 | 8.94E-87 | *ytnA* | amino acid permease YtnA |
| PP_1065 | 2.067 | 0.0001908 | *glmP* | alginate biosynthesis membrane protein |
| PP_1066 | 2.049 | 5.26E-19 | *dctD-II* | C4-dicarboxylate transport transcriptional regulator |
| PP_1067 | 2.459 | 4.75E-29 | *-* | sensor histidine kinase |
| PP_1068 | 2.519 | 3.92E-47 | *gltL* | glutamate/aspartate ABC transporter ATP binding protein |
| PP_1069 | 2.257 | 2.91E-26 | *gltK* | glutamate/aspartate ABC transporter permease |
| PP_1249 | 2.022 | 2.90E-25 | *-* | DUF4223 domain-containing protein |
| PP_1493 | 10.284 | 0 | *cheBC* | chemotaxis response regulator protein-glutamate methylesterase |
| PP_1494 | 18.988 | 0 | *-* | GGDEF domain-containing response regulator |
| PP_1502 | 2.414 | 6.47E-39 | *-* | OmpA family protein |
| PP_1566 | 2.247 | 0.00032085 | *-* | peptidase U35 |
| PP_1616 | 3.270 | 7.51E-95 | *frmA* | glutathione-dependent formaldehyde dehydrogenase |
| PP_1617 | 3.199 | 8.91E-41 | *frmC* | S-formylglutathione hydrolase/S-lactoylglutathione hydrolase |
| PP_1868 | 2.610 | 8.50E-33 | *deaD* | ATP-dependent DEAD-box RNA helicase DeaD |
| PP_1895 | 2.897 | 1.08E-29 | *yadG* | ABC transporter ATP-binding protein |
| PP_1896 | 2.553 | 1.12E-23 | *yadH* | ABC transporter permease |
| PP_2125 | 2.200 | 0.00030649 | *yegS* | lipid kinase |
| PP_2143 | 2.173 | 5.81E-09 | *lexA-I* | transcriptional repressor |
| PP_2206 | 2.121 | 1.92E-07 | *yegQ* | peptidase |
| PP_2220 | 2.428 | 1.52E-33 | *-* | DksA/TraR family C4-type zinc finger protein |
| PP_2426 | 4.213 | 4.92E-05 | *calA* | coniferyl alcohol dehydrogenase |
| PP_2453 | 9.995 | 0 | *ansB* | glutaminase-asparaginase |
| PP_2455 | 5.437 | 1.34E-180 | *rbsA-I* | ribose ABC transporter - ATP-binding subunit |
| PP_2456 | 5.011 | 6.73E-127 | *rbsC* | D-ribose ABC transporter permease |
| PP_2457 | 6.351 | 4.18E-161 | *rbsR* | DNA-binding transcriptional repressor |
| PP_2458 | 3.695 | 9.57E-83 | *rbsK* | ribokinase |
| PP_2459 | 3.882 | 6.52E-34 | *rbsD* | ribose pyranase |
| PP_2460 | 5.676 | 4.19E-198 | *Nuh* | ribonucleoside hydrolase |
| PP_2552 | 2.032 | 4.82E-08 | *-* | DOPA decarboxylase |
| PP_2561 | 2.777 | 2.34E-06 | *-* | hemolysin-type calcium-binding bacteriocin |
| PP_2584 | 2.564 | 1.04E-12 | *oguA* | 8-oxoguanine deaminase |
| PP_2631 | 11.803 | 1.47E-14 | *-* | cellulose biosynthesis protein BcsF/YhjT |
| PP_2632 | 7.689 | 2.97E-20 | *bcsG* | endoglucanase |
| PP_2634 | 12.612 | 0.00056528 | *-* | cellulose synthase |
| PP_2635 | 3.112 | 4.28E-13 | *bcsA* | cellulose synthase and translocator subunit |
| PP_2636 | 2.703 | 1.04E-10 | *bcsB* | cellulose synthase and translocator subunit |
| PP_2637 | 13.438 | 1.05E-13 | *bcsZ* | cellulose synthase endo-1,4-D-glucanase subunit |
| PP_2638 | 4.530 | 2.22E-26 | *-* | cellulose synthase operon protein C |
| PP_2645 | 3.069 | 2.67E-12 | *mgtA* | ATP-dependent magnesium transporter |
| PP_2647 | 30.251 | 9.15E-259 | *-* | MFS transporter |
| PP_2656 | 2.235 | 0.00017837 | *pstS* | phosphate ABC transporter substrate-binding protein |
| PP_2689 | 2.354 | 0.00062699 | *-* | endoribonuclease |
| PP_2824 | 2.197 | 0.011157 | *-* | TetR family transcriptional regulator |
| PP_2827 | 11.051 | 2.31E-292 | *-* | alcohol dehydrogenase |
| PP_2914 | 2.057 | 4.11E-08 | *proP* | osmosensory proline/betaine/H+ permease |
| PP_3082 | 2.166 | 0.0032971 | *-* | membrane protein |
| PP_3127 | 2.330 | 2.47E-91 | *-* | exopolysaccharide transport protein |
| PP_3148 | 2.748 | 0.00015019 | *-* | glutamine synthetase |
| PP_3260 | 2.242 | 0.010275 | *ligD* | DNA ligase D |
| PP_3346 | 2.680 | 0.0033682 | *nikE* | nickel ABC transporter ATP-binding protein |
| PP_3360 | 2.218 | 2.48E-05 | *-* | membrane protein |
| PP_3368 | 2.972 | 4.66E-09 | *-* | MFS transporter |
| PP_3424 | 2.103 | 8.93E-05 | *xcpS* | type II secretion pathway protein XcpS |
| PP_3425 | 1073.048 | 0 | *-* | RND family transporter MFP subunit |
| PP_3426 | 329.193 | 0 | *mexF* | multidrug RND transporter MexF |
| PP_3427 | 205.080 | 0 | *oprN* | multidrug RND transporter outer membrane protein OprN |
| PP_3439 | 2.418 | 3.68E-21 | *-* | AraC family transcriptional regulator |
| PP_3443 | 6.035 | 1.13E-273 | *-* | glyceraldehyde-3-phosphate dehydrogenase |
| PP_3503 | 6.009 | 3.78E-190 | *-* | sigma-54 dependent transcriptional regulator |
| PP_3519 | 32.251 | 0.00016693 | *-* | lipoprotein |
| PP_3541 | 2.027 | 0.00029597 | *-* | MgtC family transporter |
| PP_3543 | 6.546 | 0 | *-* | iron-sulfur cluster-binding protein |
| PP_3544 | 6.394 | 5.05E-264 | *-* | GntR family transcriptional regulator |
| PP_3589 | 2.764 | 3.20E-08 | *sdaC* | serine:H+ symport permease |
| PP_3593 | 2.183 | 1.38E-34 | *-* | amino acid ABC transporter substrate-binding protein |
| PP_3594 | 2.228 | 9.20E-26 | *-* | amino acid ABC transporter permease |
| PP_3595 | 2.093 | 1.15E-25 | *-* | amino acid ABC transporter permease |
| PP_3596 | 2.640 | 7.45E-55 | *amaD* | D-lysine oxidase |
| PP_3597 | 2.502 | 1.99E-36 | *-* | amino acid ABC transporter ATP-binding protein |
| PP_3613 | 2.070 | 0.00028391 | *-* | L-sorbosone dehydrogenase |
| PP_3631 | 2.025 | 5.53E-07 | *htrG* | signal transduction protein |
| PP_3635 | 2.085 | 7.60E-11 | *-* | sulfonate ABC transporter permease |
| PP_3636 | 2.050 | 3.01E-16 | *-* | sulfonate ABC transporter substrate-binding protein |
| PP_3638 | 2.147 | 9.99E-22 | *-* | acyl-CoA dehydrogenase |
| PP_3639 | 19.251 | 3.15E-182 | *-* | alkylhydroperoxidase AhpD domain-containing protein |
| PP_3661 | 2.391 | 0.00048825 | *-* | membrane protein |
| PP_3711 | 2.460 | 1.99E-08 | *-* | diguanylate cyclase |
| PP_3768 | 3.098 | 8.22E-44 | *-* | shikimate 5-dehydrogenase |
| PP_3878 | 2.257 | 1.20E-08 | *-* | minor capsid protein C |
| PP_3882 | 2.970 | 3.08E-07 | *-* | terminase small subunit |
| PP_3883 | 2.655 | 1.40E-06 | *-* | holin |
| PP_3895 | 4.435 | 0.00069978 | *-* | regulatory protein Cro |
| PP_3940 | 2.968 | 1.11E-28 | *nicT* | metabolite transport protein NicT |
| PP_3941 | 3.585 | 1.63E-57 | *nicF* | maleamate amidohydrolase |
| PP_3942 | 3.891 | 1.11E-51 | *nicE* | maleate isomerase |
| PP_3943 | 3.983 | 1.47E-36 | *nicD* | N-formylmaleamate deformylase |
| PP_3944 | 3.989 | 7.27E-69 | *nicC* | 6-hydroxynicotinate 3-monooxygenase |
| PP_3945 | 2.667 | 3.45E-56 | *nicX* | 2,5-dihydroxypyridine 5,6-dioxygenase |
| PP_3947 | 9.079 | 0.005428 | *nicA* | nicotinate dehydrogenase subunit A |
| PP_3948 | 2.873 | 7.86E-10 | *nicB* | nicotinate dehydrogenase subunit B |
| PP_4057 | 7.307 | 9.85E-30 | *-* | membrane protein |
| PP_4108 | 2.531 | 7.39E-70 | *-* | 4-aminobutyrate aminotransferase |
| PP_4284 | 2.346 | 1.26E-07 | *-* | transporter |
| PP_4288 | 2.025 | 0.0030883 | *allA* | ureidoglycolate lyase |
| PP_4297 | 2.609 | 1.64E-05 | *gcl* | glyoxylate carboligase |
| PP_4434 | 5.950 | 1.31E-35 | *dadA-I* | D-amino acid dehydrogenase small subunit |
| PP_4461 | 2.717 | 0.0030599 | *-* | MFS transporter |
| PP_4519 | 2.894 | 5.86E-139 | *tolC* | agglutination protein |
| PP_4643 | 2.647 | 4.36E-11 | *-* | xanthine/uracil permease family protein |
| PP_4838 | 2.202 | 3.57E-40 | *oprC* | copper receptor OprC |
| PP_4856 | 2.167 | 7.13E-17 | *-* | Dps family ferritin |
| PP_4983 | 2.550 | 1.97E-49 | *-* | amine oxidase |
| PP_5029 | 38.015 | 0 | *hutG* | N-formylglutamate deformylase |
| PP_5030 | 32.352 | 0 | *hutI* | imidazolonepropionase |
| PP_5031 | 27.468 | 0 | *proY* | proline (histidine) APC transporter |
| PP_5032 | 26.044 | 0 | *hutH* | histidine ammonia-lyase |
| PP_5033 | 41.840 | 0 | *hutU* | urocanate hydratase |
| PP_5035 | 4.755 | 3.75E-42 | *hutC* | histidine utilization repressor |
| PP_5036 | 30.871 | 0 | *hutF* | formiminoglutamate deiminase |
| PP_5258 | 2.027 | 5.24E-103 | *amaB* | L-piperidine-6-carboxylate dehydrogenase |
| PP_5260 | 2.318 | 2.28E-98 | *ydcJ* | metalloprotein |
| PP_5269 | 11.138 | 5.75E-39 | *dadX* | alanine racemase |
| PP_5270 | 5.722 | 1.23E-162 | *dadA-II* | D-amino acid:quinone oxidoreductase |
| PP_5602 | 2.083 | 0.001416 | *peaA* | quinohemoprotein amine dehydrogenase subunit alpha |
| PP_0681 | 3.128 | 1.16E-106 | *-* | hypothetical protein |
| PP_0831 | 3.960 | 2.34E-08 | *-* | hypothetical protein |
| PP_1503 | 2.555 | 1.28E-36 | *-* | hypothetical protein |
| PP_1511 | 2.268 | 0.0074035 | *-* | hypothetical protein |
| PP_1538 | 2.785 | 0.0041818 | *-* | hypothetical protein |
| PP_1539 | 2.645 | 0.0013668 | *-* | hypothetical protein |
| PP_1568 | 14.110 | 0.0044889 | *-* | hypothetical protein |
| PP_1691 | 4.986 | 7.96E-84 | *-* | hypothetical protein |
| PP_1810 | 3.625 | 0.012027 | *-* | hypothetical protein |
| PP_1878 | 2.445 | 1.27E-69 | *-* | hypothetical protein |
| PP_2059 | 2.102 | 9.32E-17 | *-* | hypothetical protein |
| PP_2452 | 3.159 | 0.0012547 | *-* | hypothetical protein |
| PP_2462 | 2.053 | 9.17E-45 | *-* | hypothetical protein |
| PP_2655 | 4.149 | 7.06E-10 | *-* | hypothetical protein |
| PP_2854 | 4.317 | 6.74E-16 | *-* | hypothetical protein |
| PP_2855 | 7.628 | 5.83E-27 | *-* | hypothetical protein |
| PP_2856 | 45.704 | 0 | *-* | hypothetical protein |
| PP_2857 | 94.181 | 4.26E-53 | *-* | hypothetical protein |
| PP_2858 | 29.150 | 3.72E-69 | *-* | hypothetical protein |
| PP_3036 | 4.066 | 0.00071255 | *-* | hypothetical protein |
| PP_3193 | 5.339 | 0.011106 | *-* | hypothetical protein |
| PP_3261 | 2.523 | 0.0019654 | *-* | hypothetical protein |
| PP_3392 | 6.641 | 0.011568 | *-* | hypothetical protein |
| PP_3440 | 5.801 | 1.18E-61 | *-* | hypothetical protein |
| PP_3441 | 3.281 | 3.51E-105 | *-* | hypothetical protein |
| PP_3494 | 3.438 | 0.0020173 | *-* | hypothetical protein |
| PP_3524 | 2.008 | 0.00014182 | *-* | hypothetical protein |
| PP_3542 | 2.092 | 0.00022048 | *-* | hypothetical protein |
| PP_3770 | 53.335 | 0 | *-* | hypothetical protein |
| PP_3774 | 2.818 | 1.01E-14 | *-* | hypothetical protein |
| PP_3855 | 3.298 | 3.52E-05 | *-* | hypothetical protein |
| PP_3856 | 2.217 | 5.68E-08 | *-* | hypothetical protein |
| PP_3874 | 2.327 | 0.00058416 | *-* | hypothetical protein |
| PP_3886 | 2.287 | 0.0017903 | *-* | hypothetical protein |
| PP_3901 | 2.539 | 6.98E-31 | *-* | hypothetical protein |
| PP_3928 | 5.594 | 8.81E-05 | *-* | hypothetical protein |
| PP_4435 | 4.795 | 1.34E-136 | *-* | hypothetical protein |
| PP_4858 | 70.390 | 3.20E-148 | *-* | hypothetical protein |
| PP_5034 | 3.711 | 6.38E-14 | *-* | hypothetical protein |
| PP_5191 | 2.175 | 9.82E-05 | *-* | hypothetical protein |
| PP_5430 | 3.929 | 6.40E-30 | *-* | hypothetical protein |
| PP_5435 | 2.313 | 0.00020438 | *-* | hypothetical protein |
| PP_5462 | 2.957 | 1.58E-74 | *-* | hypothetical protein |
| PP_5474 | 2.888 | 0.000161 | *-* | hypothetical protein |
| PP_5496 | 79.487 | 0 | *-* | hypothetical protein |
| PP_5524 | 2.395 | 0.0056005 | *-* | hypothetical protein |
| PP_5542 | 2.816 | 5.79E-05 | *-* | hypothetical protein |
| PP_5549 | 4.553 | 1.73E-24 | *-* | hypothetical protein |
| PP_5560 | 2.394 | 4.56E-07 | *-* | hypothetical protein |
| PP_5586 | 80.559 | 0 | *-* | hypothetical protein |
| PP_5592 | 3.287 | 0.0010095 | *-* | hypothetical protein |
| PP_5658 | 2.453 | 5.79E-16 | *-* | hypothetical protein |
| PP_5689 | 4.569 | 4.66E-07 | *-* | hypothetical protein |
