# Supplementary material for: Identification of c-di-GMP/FleQ-Regulated New Target Genes, Including cyaA, Encoding Adenylate Cyclase, in Pseudomonas putida
Source: mSystems. 2021 May 11;6(3):e00295-21. doi: 10.1128/mSystems.00295-21 (PMC8125075; doi:10.1128/mSystems.00295-21)
Supplement: TABLE S2 [file mSystems.00295-21-st002.doc]

| Gene_id | FoldChange | *P* val | Genename | Description |
| --- | --- | --- | --- | --- |
| PP_0158 | -2.736 | 7.59E-75 | *gcdH* | glutaryl-CoA dehydrogenase |
| PP_0289 | -2.723 | 1.82E-20 | *hisB* | imidazoleglycerol-phosphate dehydratase |
| PP_0290 | -2.066 | 4.01E-05 | *hisH* | imidazole glycerol phosphate synthase subunit HisH |
| PP_0292 | -2.105 | 3.76E-05 | *hisA* | phosphoribosylformimino-5-aminoimidazole carboxamide ribonucleotide isomerase |
| PP_0412 | -2.086 | 7.32E-22 | *-* | polyamine ABC transportersubstrate-binding protein |
| PP_0424 | -6.698 | 1.34E-58 | *crp* | DNA-binding transcriptional dual regulator |
| PP_0772 | -2.057 | 0.001079 | *-* | metallo-beta-lactamase family protein |
| PP_0817 | -4.499 | 1.10E-26 | *alaC* | aminotransferase |
| PP_0986 | -2.234 | 1.00E-45 | *gcvT-I* | aminomethyltransferase |
| PP_1128 | -2.314 | 1.67E-35 | *-* | OmpA family protein |
| PP_1188 | -3.489 | 3.81E-47 | *dctA-I* | C4-dicarboxylate transport protein |
| PP_1303 | -2.403 | 4.51E-15 | *cysD* | sulfate adenylyltransferase subunit 2 |
| PP_1670 | -2.138 | 1.57E-17 | *-* | lipoprotein |
| PP_1970 | -6.342 | 9.23E-06 | *-* | lipoprotein |
| PP_2041 | -2.513 | 1.78E-15 | *-* | LysR family transcriptional regulator |
| PP_2358 | -2.259 | 9.17E-53 | *-* | putative Type 1 pili subunit CsuA/B protein |
| PP_2359 | -2.405 | 5.97E-76 | *-* | putative Type 1 pili subunit CsuA/B protein |
| PP_2360 | -2.220 | 3.81E-17 | *-* | type I pili subunit CsuA/B |
| PP_2362 | -2.104 | 1.72E-51 | *-* | usher protein |
| PP_2628 | -2.374 | 2.96E-27 | *-* | ABC transporter ATP-binding protein |
| PP_2909 | -2.981 | 1.25E-53 | *csiD* | carbon starvation induced protein |
| PP_2910 | -2.767 | 6.77E-22 | *lhgO* | L-2-hydroxyglutarate oxidase |
| PP_2911 | -4.390 | 4.57E-20 | *gabP-III* | gamma-aminobutyrate permease |
| PP_3089 | -2.037 | 7.92E-101 | *hcp1* | Hcp1 |
| PP_3096 | -2.006 | 1.27E-10 | *tssG1* | TssG1 |
| PP_3097 | -2.407 | 2.94E-19 | *tssF1* | TssF1 |
| PP_3100 | -2.128 | 1.90E-11 | *tssB1* | TssB1 |
| PP_3405 | -2.139 | 1.70E-07 | *-* | membrane protein |
| PP_3455 | -2.715 | 8.96E-10 | *-* | multidrug RND transporter membrane fusion protein |
| PP_3456 | -2.179 | 1.30E-18 | *mexB* | multidrug resistance protein MexB |
| PP_3730 | -2.360 | 5.03E-08 | *aruR* | transcriptional regulator AruR |
| PP_5211 | -2.047 | 2.76E-26 | *-* | ChaC-related protein |
| PP_5222 | -2.256 | 2.54E-10 | *cyaA* | adenylate cyclase |
| PP_5298 | -2.615 | 4.71E-05 | *-* | glutamine amidotransferase |
| PP_5299 | -2.752 | 5.59E-08 | *puuA-II* | glutamate-putrescine ligase |
| PP_5338 | -2.032 | 3.58E-45 | *aspA* | aspartate ammonia-lyase |
| PP_5629 | -2.063 | 1.47E-16 | *-* | protease/amidase |
| PP_4334 | -3.380 | 7.11E-31 | *-* | ParA family protein |
| PP_4335 | -2.064 | 2.00E-13 | *-* | flagellar motor protein |
| PP_4340 | -2.671 | 5.27E-12 | *cheY* | two-component system response regulator |
| PP_4341 | -2.140 | 1.54E-23 | *fliA* | RNA polymerase sigma 28 factor |
| PP_4352 | -2.215 | 1.62E-05 | *flhB* | flagellin export apparatus substrate specificity protein |
| PP_4353 | -3.824 | 0.000378 | *fliR* | flagellar biosynthetic protein FliR |
| PP_4355 | -3.192 | 7.16E-06 | *fliP* | flagellar export apparatus protein |
| PP_4357 | -4.484 | 1.51E-13 | *fliN* | flagellar basal-body C-ring protein |
| PP_4358 | -4.592 | 1.27E-17 | *fliM* | flagellar biosynthesis switching/energizing protein |
| PP_4359 | -4.515 | 5.10E-08 | *fliL* | flagellar protein FliL |
| PP_4365 | -4.347 | 0.004266 | *fliJ* | flagellar protein FliJ |
| PP_4366 | -2.062 | 9.69E-08 | *fliI* | flagellum-specific ATP synthase |
| PP_4367 | -2.754 | 1.09E-05 | *fliH* | flagellar assembly protein FliH |
| PP_4368 | -4.554 | 6.93E-49 | *fliG* | flagellar motor switch protein |
| PP_4369 | -3.166 | 1.13E-29 | *fliF* | flagellar M-ring protein |
| PP_4370 | -6.608 | 8.27E-32 | *fliE* | flagellar hook-basal body complex protein FliE |
| PP_4371 | -2.291 | 1.48E-08 | *atoC* | two-component system DNA-binding transcriptional activator AtoC |
| PP_4374 | -2.846 | 4.81E-24 | *fliT* | flagellar protein |
| PP_4375 | -4.798 | 1.56E-25 | *fliS* | flagellar chaperone |
| PP_4376 | -3.822 | 1.08E-96 | *fliD* | flagellar filament capping protein |
| PP_4377 | -5.487 | 3.85E-14 | *-* | flagellin FlaG |
| PP_4378 | -5.254 | 1.03E-202 | *fliC* | flagellin |
| PP_4380 | -2.306 | 2.01E-17 | *flgL* | flagellar hook-associated protein FlgL |
| PP_4381 | -3.773 | 6.97E-24 | *flgK* | flagellar hook-associated protein FlgK |
| PP_4382 | -6.312 | 3.02E-11 | *flgJ* | peptidoglycan hydrolase FlgJ |
| PP_4383 | -5.471 | 2.27E-22 | *flgI* | flagellar P-ring protein |
| PP_4384 | -3.422 | 8.34E-08 | *flgH* | flagellar L-ring protein |
| PP_4385 | -5.722 | 6.40E-32 | *flgG* | flagellar basal-body rod protein FlgG |
| PP_4386 | -5.011 | 2.91E-24 | *flgF* | flagellar basal-body rod protein FlgF |
| PP_4388 | -6.086 | 1.96E-62 | *flgE* | flagellar hook protein FlgE |
| PP_4389 | -4.267 | 1.08E-09 | *flgD* | flagellar basal-body rod modification protein FlgD |
| PP_4390 | -7.510 | 0.003004 | *flgC* | flagellar basal-body rod protein FlgC |
| PP_4391 | -6.279 | 3.54E-16 | *flgB* | flagellar basal-body rod protein FlgB |
| PP_4394 | -3.073 | 1.18E-08 | *flgA* | flagella basal body P-ring formation protein |
| PP_0584 | -2.540 | 2.56E-16 | *-* | methyl-accepting chemotaxis transducer |
| PP_0802 | -2.440 | 1.99E-06 | *-* | chemotaxis protein |
| PP_1371 | -3.790 | 3.42E-34 | *pctA* | methyl-accepting chemotaxis protein PctA |
| PP_1819 | -2.426 | 1.42E-45 | *-* | methyl-accepting chemotaxis transducer |
| PP_2249 | -4.663 | 1.61E-45 | *pctB* | methyl-accepting chemotaxis protein PctB |
| PP_3557 | -2.710 | 3.38E-05 | *-* | methyl-accepting chemotaxis transducer |
| PP_4332 | -4.744 | 4.19E-25 | *-* | chemotaxis protein CheW |
| PP_4333 | -3.761 | 8.94E-20 | *-* | CheW domain-containing protein |
| PP_4337 | -2.549 | 1.95E-30 | *cheBA* | chemotaxis response regulator protein-glutamate methylesterase |
| PP_4338 | -2.336 | 3.08E-15 | *cheA* | chemotaxis histidine kinase CheA |
| PP_4888 | -2.570 | 3.07E-05 | *-* | methyl-accepting chemotaxis transducer |
| PP_5020 | -4.702 | 4.41E-48 | *-* | methyl-accepting chemotaxis protein |
| PP_0788 | -11.795 | 1.25E-66 | *-* | hypothetical protein |
| PP_1828 | -3.956 | 0.00094 | *-* | hypothetical protein |
| PP_2389 | -2.460 | 1.51E-61 | *-* | hypothetical protein |
| PP_3104 | -2.238 | 1.05E-25 | *-* | hypothetical protein |
| PP_3297 | -2.508 | 1.21E-06 | *-* | hypothetical protein |
| PP_3795 | -3.555 | 2.57E-11 | *-* | hypothetical protein |
| PP_4181 | -3.004 | 1.32E-06 | *-* | hypothetical protein |
| PP_4331 | -2.782 | 0.000492 | *-* | hypothetical protein |
| PP_4396 | -2.137 | 1.89E-25 | *-* | hypothetical protein |
| PP_4406 | -4.689 | 1.47E-11 | *-* | hypothetical protein |
| PP_4887 | -2.411 | 4.74E-41 | *-* | hypothetical protein |
| PP_5073 | -5.853 | 1.66E-32 | *-* | hypothetical protein |
| PP_5710 | -2.442 | 5.65E-40 | *-* | hypothetical protein |
