# Supplementary material for: Identification of c-di-GMP/FleQ-Regulated New Target Genes, Including cyaA, Encoding Adenylate Cyclase, in Pseudomonas putida
Source: mSystems. 2021 May 11;6(3):e00295-21. doi: 10.1128/mSystems.00295-21 (PMC8125075; doi:10.1128/mSystems.00295-21)
Supplement: TABLE S3 [file mSystems.00295-21-st003.doc]

| Gene_id | FoldChange | *P* val | Genename | Description |
| --- | --- | --- | --- | --- |
| PP_0089 | 7.479 | 3.30E-65 | *osmC* | stress-induced peroxiredoxin |
| PP_0101 | 2.286 | 1.11E-05 | *-* | sulfate transporter |
| PP_0115 | 10.864 | 3.71E-159 | *katE* | hydroperoxidase |
| PP_0233 | 2.278 | 0.00022233 | *tauA* | taurine ABC transporter substrate-binding protein |
| PP_0737 | 2.522 | 1.55E-73 | *pagL-I* | lipid A 3-O-deacylase |
| PP_1105 | 2.703 | 1.06E-09 | *-* | ATP-dependent DNA ligase |
| PP_1347 | 2.050 | 1.60E-05 | *-* | glutathione S-transferase family protein |
| PP_1502 | 6.765 | 1.02E-211 | *-* | OmpA family protein |
| PP_1516 | 2.373 | 2.00E-18 | *-* | RND membrane fusion protein |
| PP_1517 | 2.012 | 1.08E-16 | *-* | RND family transporter |
| PP_1749 | 3.456 | 2.42E-47 | *-* | acetyltransferase |
| PP_1750 | 4.190 | 3.29E-36 | *asnB* | asparagine synthetase |
| PP_1895 | 5.952 | 1.34E-40 | *yadG* | ABC transporter ATP-binding protein |
| PP_1896 | 2.928 | 5.54E-34 | *yadH* | ABC transporter permease |
| PP_2125 | 2.812 | 3.81E-08 | *yegS* | lipid kinase |
| PP_2236 | 3.157 | 4.54E-06 | *-* | alpha/beta family hydrolase |
| PP_2333 | 2.317 | 1.86E-13 | *-* | GntR family transcriptional regulator |
| PP_2377 | 3.099 | 5.76E-29 | *-* | acyltransferase |
| PP_2445 | 6.856 | 3.56E-89 | *-* | membrane protein |
| PP_2557 | 2.226 | 9.56E-05 | *-* | sensory box protein |
| PP_2558 | 2.292 | 8.81E-07 | *-* | outer membrane efflux protein |
| PP_2559 | 3.117 | 2.79E-08 | *hasE* | secretion metalloprotease |
| PP_2560 | 2.107 | 9.60E-11 | *aprDA* | alkaline protease secretion ABC transporter ATP-binding protein |
| PP_2561 | 5.810 | 5.31E-52 | *-* | hemolysin-type calcium-binding bacteriocin |
| PP_2631 | 22.439 | 4.65E-35 | *-* | cellulose biosynthesis protein BcsF/YhjT |
| PP_2632 | 15.602 | 3.45E-57 | *bcsG* | endoglucanase |
| PP_2634 | 16.615 | 6.29E-06 | *-* | cellulose synthase |
| PP_2635 | 4.771 | 2.72E-32 | *bcsA* | cellulose synthase and translocator subunit |
| PP_2636 | 4.990 | 7.60E-38 | *bcsB* | cellulose synthase and translocator subunit |
| PP_2637 | 20.357 | 2.69E-25 | *bcsZ* | cellulose synthase endo-1,4-D-glucanase subunit |
| PP_2638 | 8.648 | 2.17E-76 | *-* | cellulose synthase operon protein C |
| PP_2647 | 32.067 | 3.81E-242 | *-* | MFS transporter |
| PP_2689 | 2.221 | 0.0036679 | *-* | endoribonuclease |
| PP_2707 | 2.858 | 0.00097551 | *-* | exodeoxyribonuclease III |
| PP_2723 | 5.164 | 5.50E-13 | *yhdF* | oxidoreductase YhdF |
| PP_2725 | 8.133 | 1.25E-41 | *pfpI* | protease PfpI |
| PP_2826 | 2.241 | 5.46E-13 | *mexT* | transcriptional regulator MexT |
| PP_2827 | 14.620 | 1.66E-226 | *-* | alcohol dehydrogenase |
| PP_2914 | 3.309 | 7.28E-31 | *proP* | osmosensory proline/betaine/H+ permease |
| PP_2918 | 3.807 | 1.82E-15 | *treSA* | trehalose synthase A |
| PP_3088 | 3.183 | 7.49E-08 | *tssA1* | TssA1 |
| PP_3089 | 2.718 | 7.31E-27 | *hcp1* | Hcp1 |
| PP_3090 | 2.191 | 8.93E-46 | *tagP1* | TagP1 |
| PP_3091 | 2.746 | 4.81E-89 | *tssM1* | TssM1 |
| PP_3092 | 2.302 | 1.30E-26 | *tssL1* | TssL1 |
| PP_3093 | 2.993 | 4.87E-34 | *tssK1* | TssK1 |
| PP_3094 | 3.372 | 3.35E-38 | *tssJ1* | TssJ1 |
| PP_3095 | 2.738 | 1.45E-68 | *clpV1* | ClpV1 |
| PP_3096 | 2.494 | 3.82E-35 | *tssG1* | TssG1 |
| PP_3097 | 2.535 | 5.68E-42 | *tssF1* | TssF1 |
| PP_3098 | 2.449 | 1.81E-11 | *tssE1* | TssE1 |
| PP_3099 | 2.532 | 1.35E-43 | *tssC1* | TssC1 |
| PP_3100 | 2.833 | 1.01E-19 | *tssB1* | TssB1 |
| PP_3105 | 2.665 | 1.09E-49 | *-* | membrane protein |
| PP_3106 | 3.266 | 2.15E-44 | *vgrG1* | VgrG1 |
| PP_3107 | 4.789 | 1.83E-15 | *eagR1b* | EagR1b |
| PP_3108 | 3.245 | 9.03E-83 | *tke2* | Tke2 |
| PP_3186 | 2.025 | 0.00075919 | *-* | transcriptional regulator |
| PP_3255 | 2.785 | 2.50E-09 | *-* | non-homologous end joining protein |
| PP_3256 | 3.483 | 0.0014298 | *-* | group 2 family glycosyl transferase |
| PP_3257 | 6.729 | 2.72E-12 | *-* | putative Methyltransferase |
| PP_3259 | 2.213 | 2.57E-06 | *-* | acyl-CoA dehydrogenase-related protein |
| PP_3260 | 8.680 | 4.02E-29 | *ligD* | DNA ligase D |
| PP_3262 | 5.787 | 1.04E-06 | *-* | metallothionein |
| PP_3263 | 5.826 | 4.98E-20 | *ybhN* | phospholipid modification enzyme |
| PP_3264 | 6.979 | 1.96E-24 | *clsB* | cardiolipin synthase |
| PP_3265 | 7.158 | 1.68E-09 | *ybhP-I* | phosphohydrolase |
| PP_3267 | 2.727 | 4.43E-20 | *-* | Clp protease |
| PP_3268 | 2.357 | 1.06E-12 | *-* | MutT/nudix family protein |
| PP_3308 | 5.475 | 0.00022118 | *paoA* | aromatic aldehyde dehydrogenase 2Fe-2S subunit |
| PP_3309 | 5.473 | 7.79E-05 | *paoB* | aromatic aldehyde dehydrogenase FAD-binding subunit |
| PP_3310 | 3.113 | 1.48E-10 | *paoC* | aromatic aldehyde dehydrogenase molybdopterin-binding subunit |
| PP_3330 | 2.901 | 6.74E-09 | *-* | ferric siderophore receptor |
| PP_3360 | 6.175 | 5.59E-42 | *-* | membrane protein |
| PP_3384 | 2.285 | 0.012961 | *-* | gluconate 2-dehydrogenase gamma subunit |
| PP_3425 | 897.898 | 7.10E-155 | *-* | RND family transporter MFP subunit |
| PP_3426 | 276.409 | 4.64E-242 | *mexF* | multidrug RND transporter MexF |
| PP_3427 | 159.445 | 0 | *oprN* | multidrug RND transporter outer membrane protein OprN |
| PP_3503 | 3.556 | 9.28E-72 | *-* | sigma-54 dependent transcriptional regulator |
| PP_3519 | 21.446 | 0.0017477 | *-* | lipoprotein |
| PP_3541 | 2.404 | 3.12E-07 | *-* | MgtC family transporter |
| PP_3613 | 4.476 | 3.63E-46 | *-* | L-sorbosone dehydrogenase |
| PP_3614 | 3.890 | 1.27E-10 | *-* | membrane protein |
| PP_3762 | 2.264 | 2.81E-13 | *-* | two-component system response regulator |
| PP_3941 | 2.758 | 1.43E-33 | *nicF* | maleamate amidohydrolase |
| PP_3942 | 3.150 | 2.91E-35 | *nicE* | maleate isomerase |
| PP_3943 | 2.488 | 5.73E-15 | *nicD* | N-formylmaleamate deformylase |
| PP_3944 | 3.070 | 2.61E-41 | *nicC* | 6-hydroxynicotinate 3-monooxygenase |
| PP_3945 | 2.918 | 5.54E-62 | *nicX* | 2,5-dihydroxypyridine 5,6-dioxygenase |
| PP_3970 | 10.178 | 1.93E-13 | *ybdR* | Zn-dependent oxidoreductase |
| PP_4032 | 2.190 | 2.41E-05 | *-* | lipoprotein Blc |
| PP_4057 | 5.311 | 8.07E-25 | *-* | membrane protein |
| PP_4059 | 2.390 | 9.13E-11 | *treSB* | bifunctional trehalose synthase B/maltokinase |
| PP_4434 | 2.497 | 0.0038024 | *dadA-I* | D-amino acid dehydrogenase small subunit |
| PP_4519 | 7.296 | 1.55E-265 | *tolC* | agglutination protein |
| PP_4561 | 8.393 | 4.06E-27 | *csbD* | stress response protein |
| PP_4707 | 6.719 | 1.29E-295 | *-* | OsmY-like protein |
| PP_4735 | 2.238 | 2.11E-26 | *lldP* | L-lactate permease |
| PP_4855 | 5.352 | 3.26E-36 | *-* | osmotically-inducible lipoprotein OsmE |
| PP_4856 | 6.544 | 6.33E-41 | *-* | Dps family ferritin |
| PP_4983 | 3.045 | 1.25E-66 | *-* | amine oxidase |
| PP_5222 | 3.010 | 2.54E-44 | *cyaA* | adenylate cyclase |
| PP_5263 | 6.849 | 1.90E-54 | *-* | GGDEF domain-containing protein |
| PP_5269 | 4.009 | 2.84E-38 | *dadX* | alanine racemase |
| PP_5270 | 3.509 | 1.17E-98 | *dadA-II* | D-amino acid:quinone oxidoreductase |
| PP_5561 | 2.465 | 1.78E-14 | *tagF1* | TagF1 |
| PP_5562 | 2.029 | 9.52E-28 | *tagX1* | TagX1 |
| PP_5575 | 3.625 | 1.23E-14 | *eagR1a* | EagR1a |
| PP_0085 | 5.434 | 2.31E-41 | *-* | hypothetical protein |
| PP_0681 | 2.497 | 9.41E-55 | *-* | hypothetical protein |
| PP_0788 | 6.392 | 2.21E-193 | *-* | hypothetical protein |
| PP_0797 | 3.262 | 2.75E-28 | *-* | hypothetical protein |
| PP_1106 | 4.195 | 1.51E-20 | *-* | hypothetical protein |
| PP_1212 | 2.129 | 9.73E-09 | *-* | hypothetical protein |
| PP_1244 | 3.338 | 6.14E-61 | *-* | hypothetical protein |
| PP_1473 | 2.188 | 3.00E-28 | *-* | hypothetical protein |
| PP_1503 | 6.825 | 1.93E-188 | *-* | hypothetical protein |
| PP_1691 | 2.157 | 1.66E-15 | *-* | hypothetical protein |
| PP_1744 | 3.609 | 9.41E-99 | *-* | hypothetical protein |
| PP_1748 | 4.566 | 5.48E-30 | *-* | hypothetical protein |
| PP_1833 | 4.153 | 1.04E-42 | *-* | hypothetical protein |
| PP_1834 | 3.071 | 9.94E-39 | *-* | hypothetical protein |
| PP_1839 | 2.408 | 6.12E-31 | *-* | hypothetical protein |
| PP_2059 | 4.908 | 1.28E-51 | *-* | hypothetical protein |
| PP_2237 | 2.324 | 7.45E-10 | *-* | hypothetical protein |
| PP_2706 | 2.542 | 0.0012907 | *-* | hypothetical protein |
| PP_2722 | 7.788 | 4.30E-32 | *-* | hypothetical protein |
| PP_2828 | 2.388 | 8.16E-05 | *-* | hypothetical protein |
| PP_3104 | 3.096 | 2.57E-44 | *-* | hypothetical protein |
| PP_3153 | 2.488 | 3.15E-08 | *-* | hypothetical protein |
| PP_3195 | 2.762 | 2.03E-06 | *-* | hypothetical protein |
| PP_3240 | 2.557 | 7.43E-07 | *-* | hypothetical protein |
| PP_3241 | 11.994 | 1.54E-139 | *-* | hypothetical protein |
| PP_3251 | 2.224 | 0.00023496 | *-* | hypothetical protein |
| PP_3258 | 7.683 | 3.31E-08 | *-* | hypothetical protein |
| PP_3261 | 13.247 | 5.56E-25 | *-* | hypothetical protein |
| PP_3266 | 7.327 | 2.26E-42 | *-* | hypothetical protein |
| PP_3269 | 4.970 | 3.40E-06 | *-* | hypothetical protein |
| PP_3307 | 5.935 | 6.47E-16 | *-* | hypothetical protein |
| PP_3401 | 5.467 | 2.52E-47 | *-* | hypothetical protein |
| PP_3418 | 11.836 | 7.94E-22 | *-* | hypothetical protein |
| PP_3524 | 5.944 | 4.04E-42 | *-* | hypothetical protein |
| PP_3542 | 4.267 | 4.28E-21 | *-* | hypothetical protein |
| PP_3770 | 48.341 | 6.17E-153 | *-* | hypothetical protein |
| PP_3831 | 3.532 | 2.41E-08 | *-* | hypothetical protein |
| PP_3928 | 10.226 | 1.22E-11 | *-* | hypothetical protein |
| PP_4061 | 6.082 | 3.38E-78 | *-* | hypothetical protein |
| PP_4528 | 3.581 | 2.97E-34 | *-* | hypothetical protein |
| PP_4531 | 5.784 | 2.70E-22 | *-* | hypothetical protein |
| PP_4572 | 2.845 | 2.16E-05 | *-* | hypothetical protein |
| PP_4738 | 8.481 | 2.64E-113 | *-* | hypothetical protein |
| PP_4858 | 42.431 | 8.43E-93 | *-* | hypothetical protein |
| PP_5121 | 2.322 | 6.42E-06 | *-* | hypothetical protein |
| PP_5428 | 2.487 | 0.00088444 | *-* | hypothetical protein |
| PP_5496 | 44.679 | 5.76E-90 | *-* | hypothetical protein |
| PP_5520 | 2.496 | 2.41E-19 | *-* | hypothetical protein |
| PP_5524 | 23.189 | 3.49E-27 | *-* | hypothetical protein |
| PP_5542 | 3.496 | 1.79E-08 | *-* | hypothetical protein |
| PP_5560 | 16.782 | 3.34E-130 | *-* | hypothetical protein |
| PP_5567 | 2.125 | 9.91E-10 | *-* | hypothetical protein |
| PP_5574 | 2.609 | 3.86E-16 | *-* | hypothetical protein |
| PP_5578 | 2.894 | 2.19E-09 | *-* | hypothetical protein |
| PP_5580 | 2.843 | 6.85E-06 | *-* | hypothetical protein |
| PP_5586 | 3.473 | 5.04E-44 | *-* | hypothetical protein |
| PP_5592 | 8.070 | 5.78E-14 | *-* | hypothetical protein |
| PP_5593 | 9.741 | 3.65E-11 | *-* | hypothetical protein |
| PP_5594 | 7.179 | 0.00098179 | *-* | hypothetical protein |
| PP_5595 | 2.718 | 1.14E-10 | *-* | hypothetical protein |
| PP_5596 | 5.765 | 2.18E-146 | *-* | hypothetical protein |
| PP_5597 | 2.224 | 1.33E-14 | *-* | hypothetical protein |
| PP_5600 | 2.923 | 0.0007446 | *-* | hypothetical protein |
| PP_5608 | 9.404 | 5.68E-24 | *-* | hypothetical protein |
