# Supplementary material for: Identification of c-di-GMP/FleQ-Regulated New Target Genes, Including cyaA, Encoding Adenylate Cyclase, in Pseudomonas putida
Source: mSystems. 2021 May 11;6(3):e00295-21. doi: 10.1128/mSystems.00295-21 (PMC8125075; doi:10.1128/mSystems.00295-21)
Supplement: TABLE S4 [file mSystems.00295-21-st004.doc]

| Gene_id | FoldChange | P val | Genename | Description |
| --- | --- | --- | --- | --- |
| PP_0056 | -4.528 | 3.60E-103 | *betA-I* | choline dehydrogenase |
| PP_0057 | -5.677 | 7.60E-44 | *-* | major facilitator family transporter |
| PP_0143 | -2.095 | 4.54E-06 | *-* | membrane protein |
| PP_0166 | -2.542 | 1.36E-59 | *-* | HlyD family secretion protein |
| PP_0167 | -4.560 | 1.17E-120 | *paxB* | toxin secretion ATP-binding protein |
| PP_0168 | -9.652 | 0 | *lapA* | putative surface adhesion protein |
| PP_0173 | -10.530 | 9.40E-143 | *-* | transcriptional factor-like protein |
| PP_0241 | -2.610 | 0.00012462 | *-* | transporter |
| PP_0282 | -2.022 | 1.73E-23 | *artJ* | L-arginine ABC transporter substrate-binding subunit |
| PP_0308 | -2.297 | 1.28E-15 | *-* | dipeptidase |
| PP_0313 | -2.152 | 0.00025363 | *-* | electron transfer flavoprotein subunit beta |
| PP_0315 | -2.111 | 4.47E-17 | *gbcA* | glycine-betaine dioxygenase subunit |
| PP_0316 | -2.117 | 2.08E-22 | *gbcB* | glycine-betaine dioxygenase subunit |
| PP_0324 | -7.147 | 0.0010577 | *soxD* | sarcosine oxidase subunit delta |
| PP_0326 | -2.086 | 0.00016821 | *soxG* | sarcosine oxidase subunit gamma |
| PP_0369 | -17.820 | 5.77E-115 | *-* | diguanylate cyclase |
| PP_0445 | -2.412 | 2.86E-60 | *rplJ* | 50S ribosomal protein L10 |
| PP_0450 | -2.432 | 9.72E-37 | *rpsG* | 30S ribosomal protein S7 |
| PP_0451 | -3.964 | 1.24E-154 | *fusA* | elongation factor G 1 |
| PP_0453 | -2.036 | 4.05E-29 | *rpsJ* | 30S ribosomal protein S10 |
| PP_0454 | -2.418 | 7.24E-49 | *rplC* | 50S ribosomal protein L3 |
| PP_0455 | -3.700 | 5.44E-56 | *rplD* | 50S ribosomal protein L4 |
| PP_0456 | -2.459 | 1.47E-08 | *rplW* | 50S ribosomal protein L23 |
| PP_0457 | -2.476 | 5.07E-48 | *rplB* | 50S ribosomal protein L2 |
| PP_0459 | -2.067 | 2.49E-08 | *rplV* | 50S ribosomal protein L22 |
| PP_0460 | -2.195 | 1.55E-20 | *rpsC* | 30S ribosomal protein S3 |
| PP_0461 | -3.057 | 1.43E-38 | *rplP* | 50S ribosomal protein L16 |
| PP_0462 | -3.497 | 0.00012182 | *rpmC* | 50S ribosomal subunit protein L29 |
| PP_0463 | -3.794 | 1.54E-12 | *rpsQ* | 30S ribosomal protein S17 |
| PP_0479 | -3.496 | 4.58E-100 | *rpoA* | DNA-directed RNA polymerase subunit alpha |
| PP_0557 | -2.189 | 2.49E-06 | *acoR* | acetoin catabolism regulatory protein |
| PP_0563 | -9.068 | 5.10E-267 | *-* | two-component system response regulator |
| PP_0672 | -5.199 | 4.05E-84 | *-* | sensory box protein |
| PP_0709 | -2.206 | 1.42E-05 | *-* | NCS1 family transporter |
| PP_0768 | -5.205 | 2.13E-66 | *-* | TPR domain-containing response regulator |
| PP_0769 | -2.844 | 9.69E-08 | *-* | sensor histidine kinase |
| PP_0817 | -2.623 | 4.50E-17 | *alaC* | aminotransferase |
| PP_0914 | -2.825 | 4.49E-86 | *-* | GGDEF domain-containing protein |
| PP_1033 | -6.672 | 6.96E-99 | *-* | sulfatase domain-containing protein |
| PP_1144 | -7.690 | 5.49E-295 | *-* | membrane protein |
| PP_1206 | -3.051 | 1.34E-127 | *oprD* | basic amino acid specific porin OprD |
| PP_1297 | -2.128 | 4.22E-10 | *yhdW* | amino acid ABC transporter-binding protein YhdW |
| PP_1300 | -2.084 | 1.97E-06 | *yhdZ* | amino acid ABC transporter ATP-binding protein |
| PP_1400 | -2.247 | 0.00030974 | *kgtP* | alpha-ketoglutarate permease |
| PP_1541 | -2.178 | 0.0030871 | *-* | putative Methyltransferase |
| PP_1741 | -3.718 | 1.38E-70 | *betX* | choline/betaine/carnitine ABC transporter substrate-binding protein BetX |
| PP_1791 | -2.094 | 2.69E-29 | *-* | aldolase/synthase |
| PP_1936 | -3.520 | 6.64E-56 | *-* | membrane protein |
| PP_1970 | -9.337 | 4.30E-07 | *-* | lipoprotein |
| PP_2097 | -4.057 | 3.04E-119 | *-* | sensory box protein |
| PP_2128 | -10.741 | 2.69E-65 | *-* | CheV-like chemotaxis protein |
| PP_2357 | -2.293 | 2.86E-62 | *-* | type I pili subunit CsuB |
| PP_2358 | -2.193 | 3.02E-43 | *-* | putative Type 1 pili subunit CsuA/B protein |
| PP_2359 | -2.079 | 7.42E-44 | *-* | putative Type 1 pili subunit CsuA/B protein |
| PP_2360 | -2.457 | 3.52E-23 | *-* | type I pili subunit CsuA/B |
| PP_2362 | -2.303 | 8.46E-52 | *-* | usher protein |
| PP_2474 | -2.891 | 9.35E-15 | *-* | glutathione S-transferase family protein |
| PP_2488 | -2.090 | 7.16E-26 | *sad-I* | NAD+-dependent succinate semialdehyde dehydrogenase |
| PP_2541 | -8.467 | 5.85E-17 | *-* | transcriptional factor-like protein |
| PP_2563 | -2.383 | 3.21E-18 | *-* | antibiotic biosynthesis protein |
| PP_2862 | -5.506 | 2.83E-54 | *uppP* | undecaprenyl-diphosphatase |
| PP_2863 | -5.066 | 2.84E-16 | *-* | membrane protein |
| PP_3074 | -2.279 | 6.73E-31 | *bhbP* | D-beta-hydroxybutyrate permease |
| PP_3132 | -2.025 | 1.29E-38 | *-* | polysaccharide transporter |
| PP_3182 | -2.192 | 0.012276 | *-* | GGDEF domain-containing protein |
| PP_3378 | -2.713 | 0.0045364 | *kguK* | 2-ketogluconokinase |
| PP_3379 | -2.985 | 0.0041261 | *kguE* | epimerase |
| PP_3391 | -2.086 | 0.0021848 | *-* | tartrate MFS transporter |
| PP_3419 | -2.463 | 1.80E-21 | *-* | sigma-54 dependent transcriptional regulator/response regulator |
| PP_3420 | -2.576 | 4.81E-35 | *-* | sensor histidine kinase |
| PP_3421 | -2.730 | 2.04E-43 | *-* | sensor histidine kinase |
| PP_3453 | -2.935 | 2.28E-13 | *-* | sensor protein RstB |
| PP_3455 | -2.606 | 6.67E-10 | *-* | multidrug RND transporter membrane fusion protein |
| PP_3456 | -4.668 | 6.33E-57 | *mexB* | multidrug resistance protein MexB |
| PP_3663 | -4.980 | 2.16E-39 | *-* | GGDEF domain-containing protein |
| PP_3681 | -3.384 | 9.78E-17 | *-* | putative Helicase |
| PP_3689 | -2.913 | 4.37E-37 | *-* | serine/threonine protein phosphatase |
| PP_3691 | -3.453 | 4.91E-65 | *-* | DNA helicase-related protein |
| PP_3705 | -4.399 | 7.76E-34 | *-* | membrane protein |
| PP_3854 | -2.967 | 0.00029571 | *-* | lysozyme |
| PP_3858 | -2.042 | 0.0069141 | *-* | tail fiber protein |
| PP_3860 | -2.371 | 0.00047161 | *-* | FluMu protein gp47 |
| PP_3863 | -3.956 | 6.86E-08 | *-* | tail protein |
| PP_3864 | -3.556 | 1.00E-11 | *-* | FluMu DNA circulation protein |
| PP_3865 | -2.153 | 3.82E-08 | *-* | tail protein |
| PP_3869 | -3.728 | 1.01E-28 | *-* | sheath protein |
| PP_3878 | -2.870 | 1.30E-09 | *-* | minor capsid protein C |
| PP_3879 | -3.188 | 5.09E-14 | *-* | HK97 family portal protein |
| PP_3881 | -3.572 | 1.95E-19 | *-* | terminase large subunit |
| PP_3884 | -3.179 | 0.00029526 | *-* | lambda family holin |
| PP_4100 | -3.071 | 3.08E-19 | *-* | Cro/CI family transcriptional regulator |
| PP_4151 | -3.268 | 3.75E-10 | *-* | excisionase domain-containing protein |
| PP_4629 | -2.145 | 2.06E-17 | *-* | sensory box protein |
| PP_4742 | -2.088 | 1.12E-16 | *hsdS* | type I restriction modification system specificity protein |
| PP_4958 | -2.573 | 3.28E-10 | *ygjP* | metal-dependent hydrolase |
| PP_4959 | -3.968 | 9.14E-173 | *-* | diguanylate cyclase |
| PP_5027 | -2.026 | 1.59E-06 | *dtd* | D-tyrosyl-tRNA(Tyr) deacylase |
| PP_5033 | -2.460 | 3.39E-06 | *hutU* | urocanate hydratase |
| PP_5085 | -2.172 | 1.73E-33 | *maeB* | malic enzyme B |
| PP_5181 | -2.085 | 8.91E-55 | *spuD* | spermidine/putrescine ABC transporter substrate-binding protein |
| PP_5183 | -2.118 | 8.36E-25 | *spuB* | glutamylpolyamine synthetase |
| PP_5298 | -2.316 | 4.64E-05 | *-* | glutamine amidotransferase |
| PP_5299 | -2.208 | 5.10E-07 | *puuA-II* | glutamate-putrescine ligase |
| PP_5323 | -9.378 | 2.58E-177 | *-* | M23/M37 family peptidase |
| PP_5324 | -10.259 | 2.24E-142 | *-* | two-component system response regulator |
| PP_5491 | -3.473 | 1.13E-14 | *-* | SEC-C motif domain-containing protein |
| PP_5503 | -7.817 | 2.07E-18 | *-* | CheA signal transduction histidine kinase |
| PP_5623 | -4.581 | 2.76E-28 | *-* | membrane protein |
| PP_5665 | -2.487 | 0.0029294 | *-* | McbA family microcin B17-like processing protein |
| PP_5666 | -3.166 | 0.012289 | *-* | McbB family microcin B17-like processing protein |
| PP_0584 | -4.180 | 5.20E-35 | *-* | methyl-accepting chemotaxis transducer |
| PP_1371 | -16.494 | 2.13E-99 | *pctA* | methyl-accepting chemotaxis protein PctA |
| PP_1819 | -2.632 | 1.28E-51 | *-* | methyl-accepting chemotaxis transducer |
| PP_2249 | -11.056 | 9.62E-93 | *pctB* | methyl-accepting chemotaxis protein PctB |
| PP_2310 | -3.997 | 2.42E-189 | *-* | methyl-accepting chemotaxis transducer |
| PP_2861 | -4.848 | 4.34E-40 | *-* | methyl-accepting chemotaxis transducer |
| PP_3557 | -3.439 | 3.15E-08 | *-* | methyl-accepting chemotaxis transducer |
| PP_4888 | -4.794 | 6.37E-12 | *-* | methyl-accepting chemotaxis transducer |
| PP_5020 | -13.313 | 4.39E-105 | *-* | methyl-accepting chemotaxis protein |
| PP_4332 | -9.469 | 1.23E-48 | *-* | chemotaxis protein CheW |
| PP_4333 | -8.698 | 7.01E-45 | *-* | CheW domain-containing protein |
| PP_4334 | -6.967 | 2.09E-68 | *-* | ParA family protein |
| PP_4335 | -3.714 | 6.21E-37 | *-* | flagellar motor protein |
| PP_4336 | -4.577 | 1.03E-17 | *-* | flagellar motor rotation protein |
| PP_4337 | -4.065 | 1.30E-60 | *cheBA* | chemotaxis response regulator protein-glutamate methylesterase |
| PP_4338 | -4.977 | 5.65E-35 | *cheA* | chemotaxis histidine kinase CheA |
| PP_4339 | -2.685 | 4.38E-09 | *cheZ* | protein phosphatase CheZ |
| PP_4340 | -3.213 | 2.10E-18 | *cheY* | two-component system response regulator |
| PP_4341 | -3.179 | 3.22E-48 | *fliA* | RNA polymerase sigma 28 factor |
| PP_4342 | -2.210 | 4.05E-06 | *-* | flagellar biosynthesis regulator |
| PP_4352 | -4.352 | 4.26E-15 | *flhB* | flagellin export apparatus substrate specificity protein |
| PP_4353 | -4.247 | 1.90E-05 | *fliR* | flagellar biosynthetic protein FliR |
| PP_4355 | -29.436 | 2.02E-21 | *fliP* | flagellar export apparatus protein |
| PP_4357 | -5.044 | 1.56E-18 | *fliN* | flagellar basal-body C-ring protein |
| PP_4358 | -12.954 | 2.09E-31 | *fliM* | flagellar biosynthesis switching/energizing protein |
| PP_4359 | -9.002 | 3.00E-15 | *fliL* | flagellar protein FliL |
| PP_4365 | -3.631 | 0.0020088 | *fliJ* | flagellar protein FliJ |
| PP_4366 | -2.958 | 5.12E-14 | *fliI* | flagellum-specific ATP synthase |
| PP_4367 | -2.949 | 1.49E-10 | *fliH* | flagellar assembly protein FliH |
| PP_4368 | -8.804 | 2.90E-90 | *fliG* | flagellar motor switch protein |
| PP_4369 | -4.010 | 1.80E-44 | *fliF* | flagellar M-ring protein |
| PP_4370 | -45.445 | 8.53E-76 | *fliE* | flagellar hook-basal body complex protein FliE |
| PP_4371 | -4.258 | 1.30E-16 | *atoC* | two-component system DNA-binding transcriptional activator AtoC |
| PP_4372 | -2.489 | 2.84E-07 | *fleS* | two-component system sensor histidine kinase FleS |
| PP_4373 | -3.300 | 1.68E-101 | *fleQ* | transcriptional regulator FleQ |
| PP_4374 | -9.817 | 3.95E-80 | *fliT* | flagellar protein |
| PP_4375 | -13.338 | 1.81E-50 | *fliS* | flagellar chaperone |
| PP_4376 | -10.148 | 4.36E-205 | *fliD* | flagellar filament capping protein |
| PP_4377 | -16.439 | 1.76E-30 | *-* | flagellin FlaG |
| PP_4378 | -54.011 | 0 | *fliC* | flagellin |
| PP_4380 | -3.983 | 4.64E-42 | *flgL* | flagellar hook-associated protein FlgL |
| PP_4381 | -16.601 | 7.17E-71 | *flgK* | flagellar hook-associated protein FlgK |
| PP_4382 | -32.000 | 6.30E-23 | *flgJ* | peptidoglycan hydrolase FlgJ |
| PP_4383 | -18.594 | 1.10E-49 | *flgI* | flagellar P-ring protein |
| PP_4384 | -12.080 | 7.30E-23 | *flgH* | flagellar L-ring protein |
| PP_4385 | -27.384 | 2.31E-76 | *flgG* | flagellar basal-body rod protein FlgG |
| PP_4386 | -61.182 | 1.37E-73 | *flgF* | flagellar basal-body rod protein FlgF |
| PP_4388 | -16.645 | 3.83E-121 | *flgE* | flagellar hook protein FlgE |
| PP_4389 | -6.700 | 1.47E-16 | *flgD* | flagellar basal-body rod modification protein FlgD |
| PP_4390 | -35.569 | 5.46E-06 | *flgC* | flagellar basal-body rod protein FlgC |
| PP_4391 | -53.290 | 5.14E-40 | *flgB* | flagellar basal-body rod protein FlgB |
| PP_4394 | -9.385 | 5.41E-19 | *flgA* | flagella basal body P-ring formation protein |
| PP_4397 | -3.249 | 3.20E-20 | *ycgR* | flagellar brake protein YcgR |
| PP_4904 | -3.384 | 4.94E-33 | *motB* | flagellar motor rotation protein |
| PP_4905 | -4.478 | 3.36E-55 | *motA* | flagellar motor rotation protein |
| PP_0018 | -2.537 | 1.05E-16 | *-* | hypothetical protein |
| PP_0153 | -2.437 | 8.48E-35 | *-* | hypothetical protein |
| PP_0757 | -2.035 | 5.47E-06 | *-* | hypothetical protein |
| PP_1442 | -3.277 | 7.85E-06 | *-* | hypothetical protein |
| PP_1447 | -2.683 | 0.0059229 | *-* | hypothetical protein |
| PP_1542 | -4.107 | 4.59E-11 | *-* | hypothetical protein |
| PP_1544 | -4.327 | 2.83E-05 | *-* | hypothetical protein |
| PP_1828 | -16.106 | 9.41E-09 | *-* | hypothetical protein |
| PP_1921 | -4.625 | 7.06E-146 | *-* | hypothetical protein |
| PP_1958 | -6.148 | 3.93E-67 | *-* | hypothetical protein |
| PP_1959 | -2.759 | 9.31E-43 | *-* | hypothetical protein |
| PP_2118 | -2.143 | 8.65E-11 | *-* | hypothetical protein |
| PP_2129 | -2.159 | 1.61E-10 | *-* | hypothetical protein |
| PP_2294 | -2.098 | 1.20E-45 | *-* | hypothetical protein |
| PP_2363 | -2.358 | 9.69E-30 | *-* | hypothetical protein |
| PP_2532 | -4.073 | 5.24E-06 | *-* | hypothetical protein |
| PP_2858 | -7.298 | 0.0075249 | *-* | hypothetical protein |
| PP_2859 | -5.425 | 2.06E-08 | *-* | hypothetical protein |
| PP_3678 | -2.094 | 3.45E-15 | *-* | hypothetical protein |
| PP_3680 | -3.216 | 2.24E-12 | *-* | hypothetical protein |
| PP_3690 | -3.107 | 1.52E-09 | *-* | hypothetical protein |
| PP_3692 | -14.236 | 0 | *-* | hypothetical protein |
| PP_3706 | -4.709 | 1.18E-43 | *-* | hypothetical protein |
| PP_3795 | -6.312 | 2.26E-22 | *-* | hypothetical protein |
| PP_3855 | -2.668 | 0.0060487 | *-* | hypothetical protein |
| PP_3856 | -2.995 | 2.38E-06 | *-* | hypothetical protein |
| PP_3871 | -3.303 | 1.38E-05 | *-* | hypothetical protein |
| PP_3873 | -2.827 | 8.15E-05 | *-* | hypothetical protein |
| PP_3874 | -3.281 | 0.00098222 | *-* | hypothetical protein |
| PP_3875 | -4.049 | 1.85E-07 | *-* | hypothetical protein |
| PP_3877 | -4.475 | 2.06E-37 | *-* | hypothetical protein |
| PP_3904 | -2.543 | 6.50E-93 | *-* | hypothetical protein |
| PP_4331 | -4.178 | 3.27E-07 | *-* | hypothetical protein |
| PP_4406 | -8.016 | 1.22E-20 | *-* | hypothetical protein |
| PP_4447 | -3.392 | 3.75E-96 | *-* | hypothetical protein |
| PP_4448 | -12.111 | 0 | *-* | hypothetical protein |
| PP_4535 | -2.312 | 6.22E-17 | *-* | hypothetical protein |
| PP_4955 | -6.021 | 7.68E-43 | *-* | hypothetical protein |
| PP_5073 | -3.019 | 9.72E-21 | *-* | hypothetical protein |
| PP_5129 | -2.781 | 3.06E-55 | *-* | hypothetical protein |
| PP_5172 | -4.163 | 0.0024728 | *-* | hypothetical protein |
| PP_5274 | -2.013 | 0.0010752 | *-* | hypothetical protein |
| PP_5395 | -2.291 | 3.58E-64 | *-* | hypothetical protein |
| PP_5429 | -7.181 | 5.45E-65 | *-* | hypothetical protein |
| PP_5430 | -6.020 | 3.72E-15 | *-* | hypothetical protein |
| PP_5449 | -5.294 | 5.90E-23 | *-* | hypothetical protein |
| PP_5462 | -2.611 | 5.86E-28 | *-* | hypothetical protein |
| PP_5463 | -2.067 | 5.16E-09 | *-* | hypothetical protein |
| PP_5492 | -4.685 | 2.61E-33 | *-* | hypothetical protein |
| PP_5516 | -10.346 | 2.78E-30 | *-* | hypothetical protein |
| PP_5517 | -7.423 | 5.13E-192 | *-* | hypothetical protein |
| PP_5540 | -2.199 | 1.58E-34 | *-* | hypothetical protein |
| PP_5549 | -2.004 | 0.00052421 | *-* | hypothetical protein |
| PP_5624 | -4.460 | 1.53E-85 | *-* | hypothetical protein |
| PP_5638 | -3.549 | 0.0022605 | *-* | hypothetical protein |
| PP_5641 | -3.659 | 1.24E-70 | *-* | hypothetical protein |
| PP_5645 | -3.165 | 6.28E-40 | *-* | hypothetical protein |
| PP_5696 | -2.055 | 3.73E-06 | *-* | hypothetical protein |
| PP_5709 | -3.543 | 9.63E-11 | *-* | hypothetical protein |
| PP_5710 | -39.197 | 3.70E-194 | *-* | hypothetical protein |
| PP_5737 | -4.082 | 1.21E-71 | *-* | hypothetical protein |
| PP_5738 | -2.371 | 7.58E-36 | *-* | hypothetical protein |
