# Supplementary figures and images for: Identification of c-di-GMP/FleQ-Regulated New Target Genes, Including cyaA, Encoding Adenylate Cyclase, in Pseudomonas putida
Source: mSystems. 2021 May 11;6(3):e00295-21. doi: 10.1128/mSystems.00295-21 (PMC8125075; doi:10.1128/mSystems.00295-21)

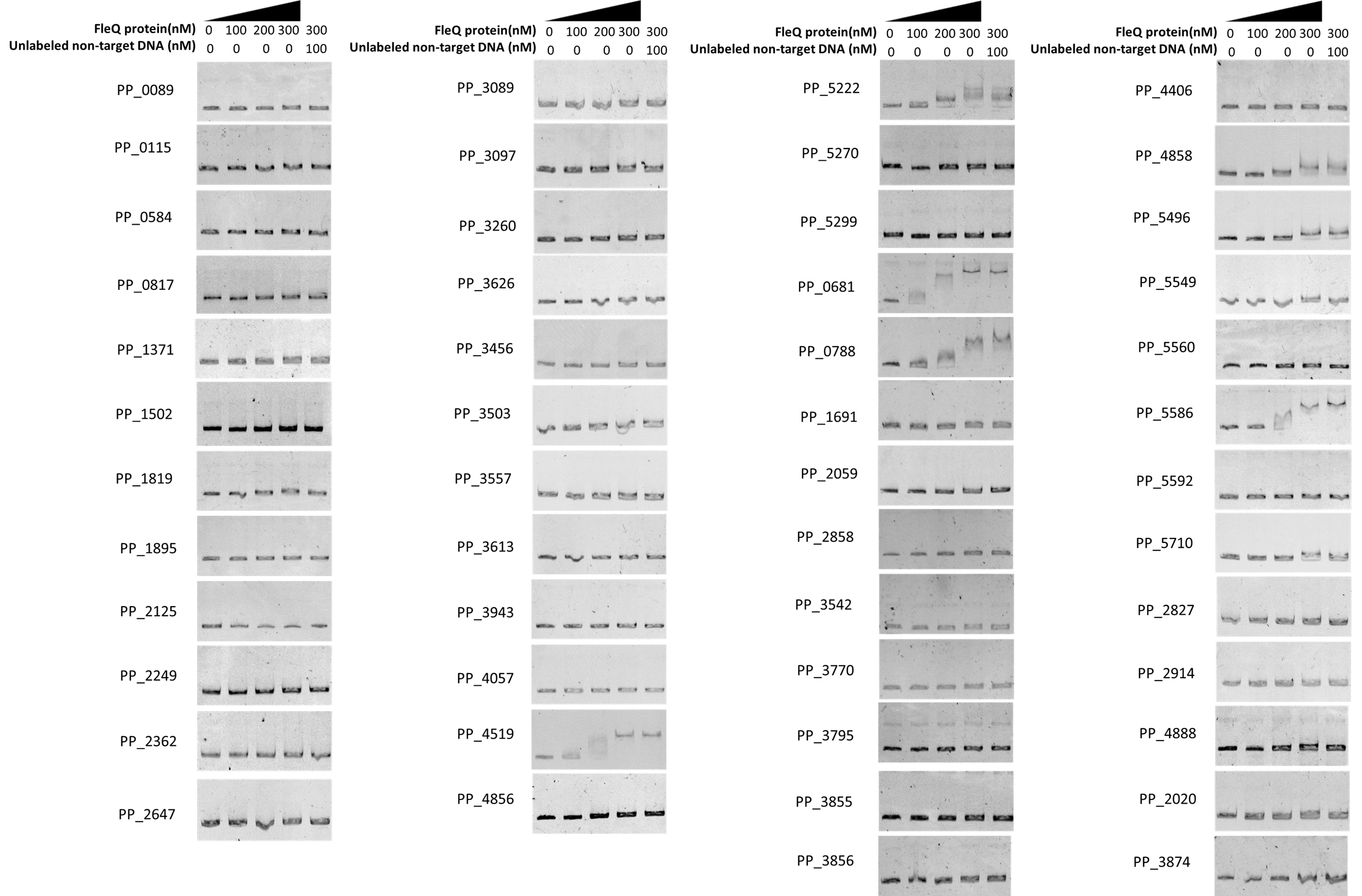

Supplement: FIG S1 [file mSystems.00295-21-sf001.tif]

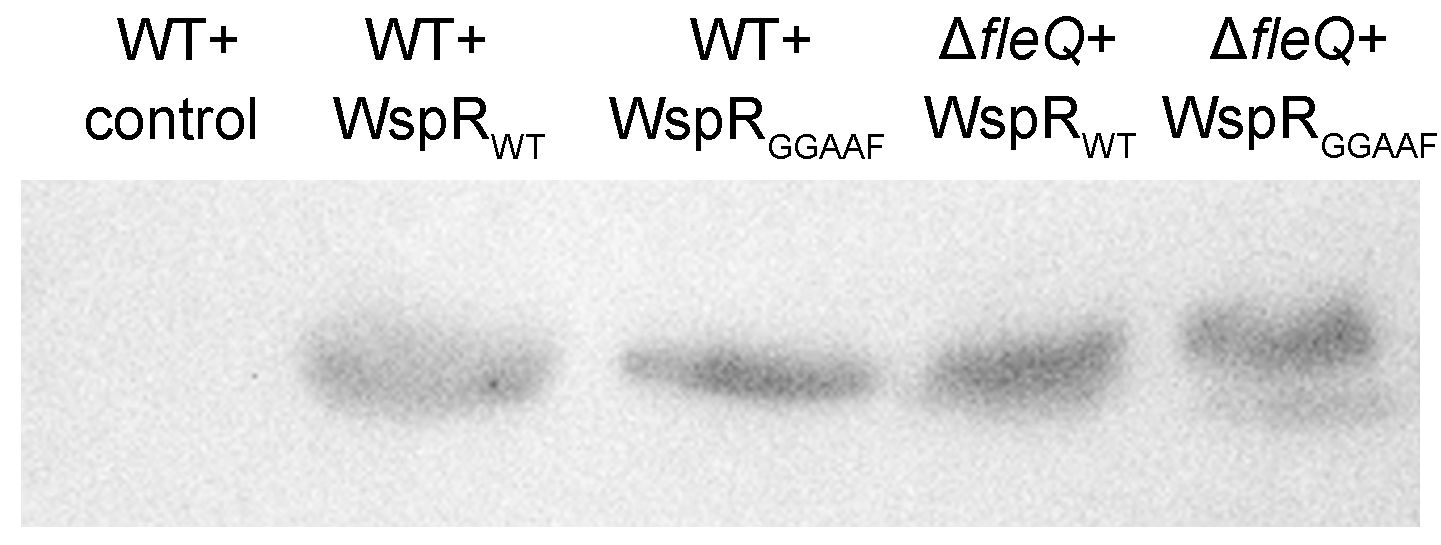

Supplement: FIG S2 [file mSystems.00295-21-sf002.tif]

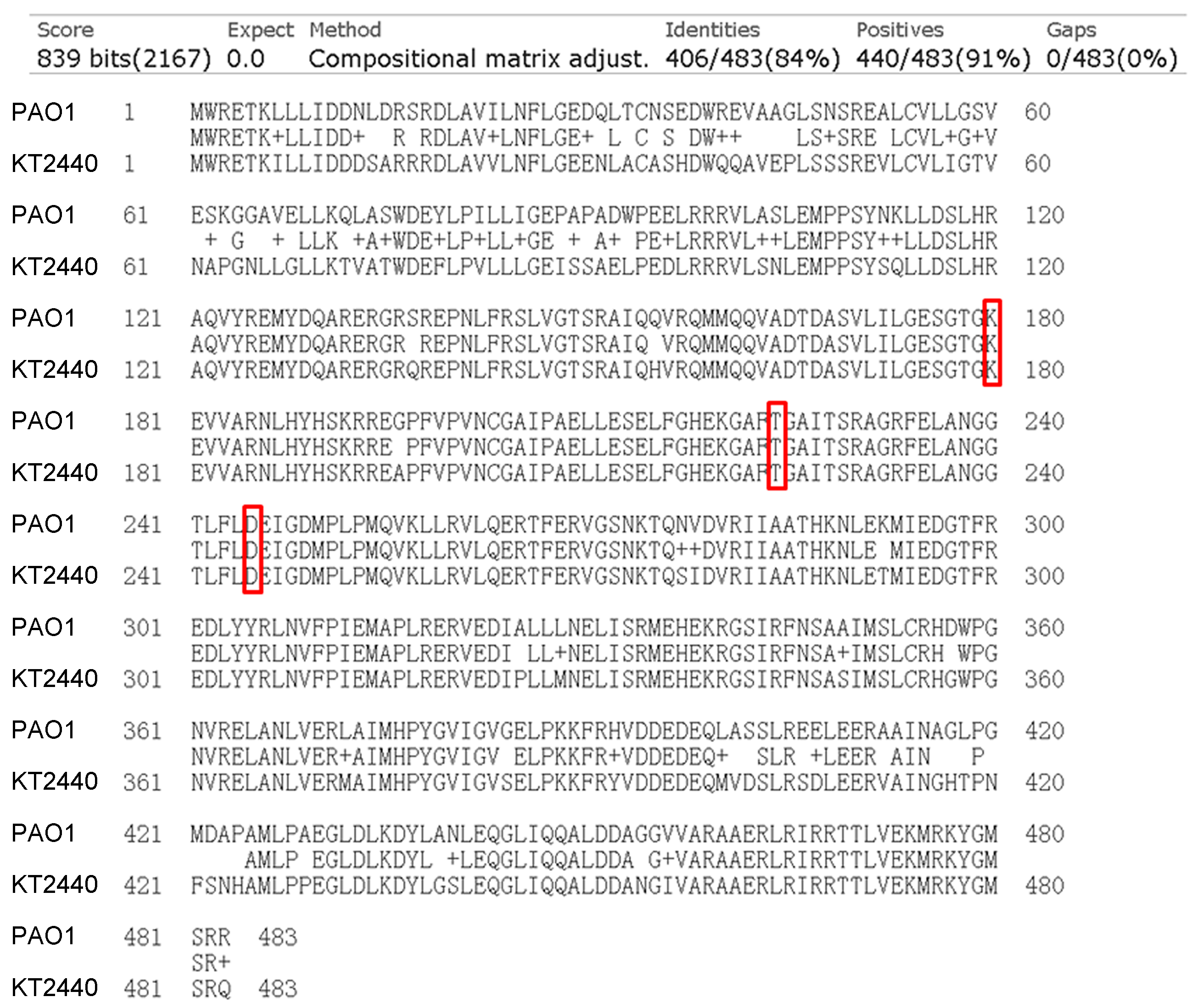

Supplement: FIG S3 [file mSystems.00295-21-sf003.tif]
