# Supplementary material for: Identification of c-di-GMP/FleQ-Regulated New Target Genes, Including cyaA, Encoding Adenylate Cyclase, in Pseudomonas putida
Source: mSystems. 2021 May 11;6(3):e00295-21. doi: 10.1128/mSystems.00295-21 (PMC8125075; doi:10.1128/mSystems.00295-21)
Supplement: TABLE S5 [file mSystems.00295-21-st005.doc]

| Strains/plasmids | Description | Source or reference |
| --- | --- | --- |
| *E. coli* strains  DH5α | λ-Φ80dlacZΔM15Δ(lacZYA-argF)U196recA1endA1 hsdR17(rK- mK -) supE44 thi-1 gyrA relA1 | Invitrogen Corp |
| S17-1/λpir | RK2 tra regulon, pir, host for pir-dependent plasmids | Invitrogen Corp |
| XL1-Blue MRF' Kan | ∆(mcrA)183, ∆(mcrCB-hsdSMR-mrr)173, endA1, supE44, thi-1, recA1, gyrA96, relA1, lac [F´proAB, lacI^q^Z∆M15, Tn5 (Kan^R^)] | Stratagene |
| BL21(DE3) | F-，ompT，hsdS (rBB-mB－)，gal，dcm (DE3) | Invitrogen Corp |
| *P. putida* strains |  |  |
| WT KT2440 | Wild-type KT2440 | Lab stock |
| WT+control | Wild-type KT2440 containing pBBR1-MCS5 | This work |
| WT+*wspR* | Wild-type KT2440 containing pBBR1-MCS5-*wspR* | This work |
| Δ*fleQ+*control | *fleQ* mutant containing pBBR1-MCS5 | This work |
| Δ*fleQ+wspR* | *fleQ* mutant containing pBBR1-MCS5-*wspR* | This work |
| Δ*fleQ*Δ*bifA* | *fleQ* and *bifA* double deletion mutant | This work |
| cΔ*fleQ* | Complemented *fleQ* mutant with pBBR1-MCS5-*fleQ* | This work |
| cΔ*fleQ*_K180A_ | *fleQ* mutant complemented with point mutated *fleQ*(K180A) | This work |
| cΔ*fleQ*_D245A_ | *fleQ* mutant complemented with point mutated *fleQ* (D245A) | This work |
| cΔ*fleQ*_T224S_ | *fleQ* mutant complemented with point mutated *fleQ* (T224S) | This work |
| WT-*lapA*Ω3×HA | Wild type with a 3×HA tag fused into *lapA* gene | This work |
| Δ*lapE+*control | *lapE* deletion mutant containing pBBR1-MCS5, *lapA*Ω3×HA | This work |
| cΔ*lapE* | Complemented *lapE* mutant with pBBR1-MCS5-*lapE*, *lapA*Ω3×HA | This work |
| Δ*lapE+wspR* | *lapE* deletion mutant containing pBBR1-*wspR*, *lapA*Ω3×HA | This work |
| Δ*fleQ*-*lapA*Ω3×HA | *fleQ* mutant with a 3×HA tag fused into *lapA* gene | This work |
| Δ*fleQ*Δ*lapE*-*lapA*Ω3×HA | *fleQ* +*lapE* mutant with a 3×HA tag fused into *lapA* gene | This work |
| Plasmids |  |  |
| pBBR1-401 | Knockout vector, derived from pBBR1-MCS5, with origin fragment replaced by ori R6K origin fragment | Lab stock |
| pBBR401-XUP-kan-XDW | Suicide plasmid containing up and down homologous region of target gene X | This work |
| pBBR–*lacZ* | Derived from pBBR1-MCS5, harbors a promoterless *lacZ* gene, Gm^r^, Tet^r^ | Lab stock |
| pBBR-*X-*pro-*lacZ* | Reporter plasmid constructed by ligating target promoter DNA to the promoterless *lacZ* gene in pBBR–*lacZ*, Gm^r^, Tet^r^ | This work |
| p*CdrA*::*gfp*^C^-tet | a derivate of p*CdrA*::*gfp*^C^, with its gentamicin resistance gene replaced by a tetracycline resistance gene, Tet^r^ | Lab stock |
| pTnmod-RKm | Km^r^, plasposon, oriR6K replicon | Lab stock |
| pBBR1-MCS5 | Expression vector. Gm^r^, Mob^+^ | Lab stock |
| pBBR1MCS5-*fleQ* | Complete *fleQ* gene and its own promoter in pBBR1-MCS5, Gm^r^ | This work |
| pBBR1MCS5-*lapE* | Complete *lapE* gene and its own promoter in pBBR1-MCS5, Gm^r^ | This work |
| pBBR1MCS5-*wspR* | Complete *wspR* gene and its own promoter in pBBR1-MCS5, Gm^r^ | This work |
| pTRG-*fleQ* | Complete *fleQ* gene in pTRG vector, Tet^r^ | This work |
| pBXcmT-xpro | Reporter plasmid constructed by ligating target promoter DNA to pBXcmT, Cm^r^ | This work |
| pBBR1MCS5-*lapE*-*gfp* | Expression vector containing *lapE* promoter and *lapE*::*gfp* fusion, Gm^r^ | This work |
| pBBR1MSC5-*cyaA*-*gfp* | Expression vector containing *cyaA* prmoter and cyaA::*gfp* fusion, Gm^r^ | This work |
