# Supplementary material for: Identification of c-di-GMP/FleQ-Regulated New Target Genes, Including cyaA, Encoding Adenylate Cyclase, in Pseudomonas putida
Source: mSystems. 2021 May 11;6(3):e00295-21. doi: 10.1128/mSystems.00295-21 (PMC8125075; doi:10.1128/mSystems.00295-21)
Supplement: TABLE S6 [file mSystems.00295-21-st006.doc]

| Primers | Sequence^a^ |
| --- | --- |
| *lapA*_UpS | CATGCCATGGTCAACTCGGCGGTAGACG |
| *lapA*_UpA | CCGGAATTCCTGTTCGAACTTGCCAACA |
| *lapA*_DwS | CGCGGATCCCTGACACTGGACAAGACCC |
| *lapA*_DwA | TGCTCTAGAGACGCTGCCATCGGACT |
| *lapE*_UpS | CCGCTCGAGTCAAGCCACGCCACCAC |
| *lapE*_UpA | CCGGAATTCGCCAGGGCCAACAGTAAAG |
| *lapE*_DwS | CGCGGATCCTATTTCGTTGCCACCAGAGG |
| *lapE*_DwA | TGCTCTAGAAGACCGCCATGCCCAGA |
| *kan*S | CGCGGATCCCCTCGTGAAGAAGGTGTTG |
| *kan*A | CCGGAATTCAAGCCACGTTGTGTCTCAA |
| *clapES* | CCGCTCGAGCCCTCAAGGGCAAGGTCTA |
| *clapEA* | CCGGAATTCCCATTCGCAGCACAAAGC |
| *cyaA*proS | CGGGGTACCTCGGTGAACAGGTTGCGC |
| *cyaA*proA | CGGGGTACCGGTCGATGCCTTCGTCCA |
| *fleQs* | CGCGGATCCATGTGGCGTGAAACCAAG |
| *fleQa* | CCGGAATTCGCGTTAAGACCGAGAAGG |
| *lapEps* | CGCGGATCCCTCAAGGGCAAGGTCTACGC |
| *lapEpa* | CGCGGATCCCCAGGGCCAACAGTAAAGCA |
| *cyaAps* | CGCGGATCCCCATGTTGCGCTCGTCG |
| *cyaAps* | CGCGGATCCGCGGATTTCGTGAGGGTG |
| *0681ps* | CGCGGATCCTCCACGTCTACGCGGTCAG |
| *0681pa* | CGCGGATCCCTGCATTCGATACGGGTCA |
| *0788ps* | CGCGGATCCTTTTCACGGGTGATGATGG |
| *0788pa* | CGCGGATCCCCTCGGTCGATGGTTGC |
| *4858ps* | CGCGGATCCTCGCCAGCACGGTAAGC |
| *4858pa* | CGCGGATCCGAAGGGACAGGGTCAGGGT |
| *5496ps* | CGCGGATCCGGTGGCTTGCACCTGTCG |
| *5496pa* | CGCGGATCCCCGATGCGGAAATGGC |
| *5586ps* | CGCGGATCCGAACACCAGGGCGAAGAGT |
| *5586pa* | CGCGGATCCTAGCATCACCAGCAAGAACAT |
| Qpcr0089S | AACCCTTATGGCTTCAACAC |
| Qpcr0089A | GATCCGCTCTGCGGTAAG |
| Qpcr0115S | CTCGGCTGTTCTTCCATA |
| Qpcr0115A | TATGTTCACGCTCCACTT |
| Qpcr0817S | TCAAAGGGCTGCACGAACT |
| Qpcr0817A | TTGCGAGCCAAACCATCA |
| Qpcr1502S | ACGCCTACCCGAACATCAC |
| Qpcr1502A | GCCATAGCCCTGGGAAAC |
| Qpcr1895S | CGTGGTGCCGCAAGAGT |
| Qpcr1895A | GGGTTCGTCCAGGATCAGC |
| Qpcr1970S | AGACGCTGTTGATTCCAT |
| Qpcr1970A | ACAAGATATTACCTTCGCCTAT |
| Qpcr2125S | GGCAAGCAGGCGATGAAC |
| Qpcr2125A | CCGTACCCAAGGGCAACAA |
| Qpcr2362S | TCAAACTGGGTAGCCGTGTC |
| Qpcr2362A | CCAACGGACCAATCAAAGG |
| Qpcr2561S | TTCGGTGTCCTACTACTG |
| Qpcr2561A | CCTGATAGATCGCCTTGA |
| Qpcr2647S | CCCGTTGATTTCCGTAGG |
| Qpcr2647A | GAAGCCGCCAATACTGATA |
| Qpcr2689S | CCCGGAACTGAGCAGTGAA |
| Qpcr2689A | CTGAGCCTTTGCCAGGTATGT |
| Qpcr2827S | CAGATGTCACTGTTGGGT |
| Qpcr2827A | ACTGAATGTTCTTCTGGAATG |
| Qpcr2914S | TGCTCAGCGACAAGTTTGG |
| Qpcr2914A | GTAACGGATGTGCGTGGG |
| Qpcr3089S | CGTTGCCCTGAGCGACTT |
| Qpcr3089A | GCGGTTCTGCTGGGTGTA |
| Qpcr3097S | TACTCCGAACGCTCATTC |
| Qpcr3097A | CGAAGAACATGAACTTGTCA |
| Qpcr3260S | TGCCAGCACAGTTACAGCC |
| Qpcr3260A | TCAGCCACCACCATTTCG |
| Qpcr3360S | TATTCGGCATCTCCATTCT |
| Qpcr3360A | CATTATCTGGCTCAGTGG |
| Qpcr3426S | CAACCTTGGCGAGATGAT |
| Qpcr3426A | TGATGAAGCCGTTGTAGT |
| Qpcr3456S | TGTTGGCGACCCTCACCTC |
| Qpcr3456A | CCCGTTCGGAGCCATACAA |
| Qpcr3503S | CAACGCAGGCTGGGATGT |
| Qpcr3503A | CGGCAGGGTATGGAAGTCG |
| Qpcr3519S | CACCCTTGTCGCTTCTGCC |
| Qpcr3519A | CGCCGTTGCTGCTCGTAA |
| Qpcr3541S | GCGAGGTGACGCAAATCC |
| Qpcr3541A | CACCCGACTGAGTGCCTGT |
| Qpcr3613S | TTGCTGAGTGCCTGTGGTG |
| Qpcr3613A | CCTTGGGCTTGAGCTTGG |
| Qpcr3878S | CCCGAACGAGCCGATTA |
| Qpcr3878A | GAGGTTGTCCACCGCAAG |
| Qpcr3943S | GGCATTATTTCGACACCTAT |
| Qpcr3943A | GAATATCCGCAGCACAAG |
| Qpcr4057S | TCAGTGCCAGTGGGTTGAGT |
| Qpcr4057A | AGCTGCTTTGCAGGTTGTTG |
| Qpcr4434S | GCAGGGCAGGTTTCTCC |
| Qpcr4434A | CCATCCGTTCTTTGTTGACT |
| Qpcr4519S | ATTACCGAGGCTGTCCAA |
| Qpcr4519A | GTTTCCTTGTTGTGGTTTCC |
| Qpcr4856S | TGTGGCTGCCGAGGAGT |
| Qpcr4856A | CAGGTCTTCCAGCACCATCT |
| Qpcr4983S | TTACGAGGTCAAGCGATA |
| Qpcr4983A | GTATTTACTGATCTGCCCTG |
| Qpcr5033S | CGATTACGGCAACAACAT |
| Qpcr5033A | TCAGTTCCTTGACCTTGG |
| Qpcr5222S | CCGTTCGTTGAGTTGTTC |
| Qpcr5222A | AGCTTCTCGAAAGCCTTG |
| Qpcr5270S | GCATCAATGGCGTGTGGA |
| Qpcr5270A | GAATAGCCTTTCAGCGGATAC |
| Qpcr5299S | CCGACGCCATCGTCAAG |
| Qpcr5299A | TTTCCAGCACGCCACCA |
| Qpcr0584S | AGACATTGCCCAAGGTGAGG |
| Qpcr0584A | CGAATGGCACCGAGGAAC |
| Qpcr1371S | ATCGTGATACTCGCCTTC |
| Qpcr1371A | CCATTTCAGCCAGATAGTTT |
| Qpcr1819S | GTGAGCCAGGAGCGTATTGC |
| Qpcr1819A | CGCCACTTCGCCCAACA |
| Qpcr2249S | TCATTGGCTCAAGGTCTC |
| Qpcr2249A | GATATTCTGGCGGATGGTA |
| Qpcr3557S | GGCGGTCAACGGCAAGA |
| Qpcr3557A | CGCCTTTGGTCGGGTCTA |
| Qpcr4888S | CGAGCCTGATTGAGAACCTTG |
| Qpcr4888A | ACTGCTTCCTTGGCACCTTT |
| Qpcr5020S | GTATCAATGGTTAGCCCAATC |
| Qpcr5020A | CGAGATGTTTCCCAGTTTG |
| Qpcr0681S | TGACAACGGCATGTGGACG |
| Qpcr0681A | GACGATGGCATTGAGCACC |
| Qpcr0788S | GCTCAAGGTGGCATTACT |
| Qpcr0788A | CTGGACGATACGCTCTAC |
| Qpcr1691S | TTGAAGTGCCTTATCGTTGC |
| Qpcr1691A | ATGGCGGCTGTACTGCTC |
| Qpcr1828S | TGTGCAGTTCGGCTATGCC |
| Qpcr1828A | CAGGCGGTTCTGCTCTTTGT |
| Qpcr2059S | GAAGCCGAAGGCGATGC |
| Qpcr2059A | CCTTGACGAACGAGGACACC |
| Qpcr2858S | CCTATTACTGGTGGGTTGT |
| Qpcr2858A | TTGGCATTGATGGTGGTA |
| Qpcr3524S | GCACCACGGCAATGAGG |
| Qpcr3524A | GCACCACGGCAATGAGG |
| Qpcr3542S | CTGATCGGCGAAGACCTGC |
| Qpcr3542A | GCCTCGGTTTCGGTAATGG |
| Qpcr3770S | ATCCAGATCATGTCCGTTGT |
| Qpcr3770A | CCGTTGCGAACCAGGTAC |
| Qpcr3795S | CATCACGGCAAGCAACCT |
| Qpcr3795A | GGCAGCCTCGTCCATCTT |
| Qpcr3855S | CCTCAGTAACGCCTACGACG |
| Qpcr3855A | TTCGCAATGATCGACACCTT |
| Qpcr3856S | TGCCAGCAGTTCGGTGTT |
| Qpcr3856A | GGGCGAGGCTTAGAGTCTTC |
| Qpcr3874S | CGTTGGCACAGCGGTCTA |
| Qpcr3874A | TCCCGCCACCACCTCAT |
| Qpcr3928S | TTGAATCGCCAGCACCAC |
| Qpcr3928A | TTGAATCGCCAGCACCAC |
| Qpcr4406S | GCGACCACCGAGACGAT |
| Qpcr4406A | TCCACCTTGGCGACCTG |
| Qpcr4858S | CTGACCCTGTCCCTTCTG |
| Qpcr4858A | CCTTCAGCTACGGTGTTC |
| Qpcr5073S | TATCTCGCCCTCATCCTCG |
| Qpcr5073A | GTCGTCGTTGTTGATCTTCTTG |
| Qpcr5430S | AGATGACCGACGAGTGCG |
| Qpcr5430A | GGATTCTTGCCTTCTTGCA |
| Qpcr5462S | TTCTATGCCGCTACCAC |
| Qpcr5462A | ACCTGCCGACCTTGTT |
| Qpcr5496S | CTTCAATCTCTTCCCGGTTAT |
| Qpcr5496A | AGGTACTGCTCGGTGTAG |
| Qpcr5524S | GCGGCGTATCGGTATCAG |
| Qpcr5524A | GGCGTTGCCGAACACTT |
| Qpcr5542S | GAGCCTTCAGTCCACCAG |
| Qpcr5542A | ACCTCGACCAACATCCC |
| Qpcr5549S | TATCGTTCCCGCTGTGC |
| Qpcr5549A | CCAACTCATCCCGCTTG |
| Qpcr5560S | ATTCGCCCGCTGTCCCT |
| Qpcr5560A | GCCGGATTGGTCAGTTTGC |
| Qpcr5586S | ATGTTCTTGCTGGTGATG |
| Qpcr5586A | AGGAATGATACTGTTCTGATT |
| Qpcr5592S | ACTTACCCGCCTGCTGC |
| Qpcr5592A | CGCCACGCTACCGTCAT |
| Qpcr5710S | ACTGCGTCATACGGCTTAG |
| Qpcr5710A | AGCCTGCACCGGAAACT |
| rpoD-F | CCTGATCCAGGAAGGCAACAT |
| rpoD-R | CAGGTGGCATAGGTCGAGAACT |
| fleQ-K180A1F | CCGGAATTCCGGGCCAATCTGGAAGC |
| fleQ-K180A1R | GCGCGCTACCACTTCCGCGCCGGTGCCGGACTC |
| fleQ-K180A2F | GAGTCCGGCACCGGCGCGGAAGTGGTAGCGCGC |
| fleQ-K180A2R | CGCGGATCCAAACCTCAAGGCGAACACC |
| wspR-AA1F | CCGCTCGAGGCAGCCAGGGCAGGTACT |
| wspR -AA1R | CAGCACCAGGGCAAACGCTGCGCCGCCATAGCGCGC |
| wspR -AA2F | GCGCGCTATGGCGGCGCAGCGTTTGCCCTGGTGCTG |
| wspR -AA2R | CCGGAATTCCGTATGTCGATAGGGCAACC |
| EMSA0089S | TGTCAAGCTACCTGCTGACATGGTTGGGCAGCATCA |
| EMSA0089A | GCGCCGCTTTCGGTAGA |
| EMSA0115S | TGTCAAGCTACCTGCTGATCGGCGACGGTCTTGGA |
| EMSA0115A | CTGGGCATGATCGGCTCT |
| EMSA0584S | TGTCAAGCTACCTGCTGATGCTGACCTGGCGGATTT |
| EMSA0584A | CAAGTTGCTGACCTTGTGGC |
| EMSA0817S | TGTCAAGCTACCTGCTGAGAGGACGACAGCAAGTGG |
| EMSA0817A | TCGGCAGTGATGTTGAAGAC |
| EMSA1371S | TGTCAAGCTACCTGCTGATCAGCCACCTTGCCCTGT |
| EMSA1371A | AGGCGAGTATCACGATCAGTG |
| EMSA1502S | TGTCAAGCTACCTGCTGAGATAACGACGATGTCCGTAGCA |
| EMSA1502A | CCGCCCTTGAAGAACTGG |
| EMSA1819S | TGTCAAGCTACCTGCTGATGGCACAGTAAACAGCAGCAA |
| EMSA1819A | CAGCAGCACCAAGGCAAA |
| EMSA1895S | TGTCAAGCTACCTGCTGAGCACGGAGCAGGGCTGGTAT |
| EMSA1895A | TCGGATGGACAGGGCGGAAC |
| EMSA2125S | TGTCAAGCTACCTGCTGACAGGTTGAGTGGGCTGTGC |
| EMSA2125A | CTTCGTTCATCGCCTGCTT |
| EMSA2249S | TGTCAAGCTACCTGCTGAAGAGCGTGAACAGGGCAAAT |
| EMSA2249A | ACACTGGTCCACCGACAGC |
| EMSA2362S | TGTCAAGCTACCTGCTGATGCAGGTCAAAGCCCTCG |
| EMSA2362A | GGTACTGCCGCTGAACAACA |
| EMSA2647S | TGTCAAGCTACCTGCTGATGACCAGGGCGAAAGCG |
| EMSA2647A | TGCCGAGCAGGAACAGGA |
| EMSA2827S | TGTCAAGCTACCTGCTGAGGTGACAGCAGGGCGAAGA |
| EMSA2827A | AAACGGATCATGCGGGAC |
| EMSA2914S | TGTCAAGCTACCTGCTGACGAAGCGGTGCGTGAGT |
| EMSA2914A | AGGCCGATTGGCTGGAC |
| EMSA3089S | TGTCAAGCTACCTGCTGAGATGCCGTCGATTTGGATA |
| EMSA3089A | GCAAGCTGCTGAACACCC |
| EMSA3097S | TGTCAAGCTACCTGCTGAGTCATTGGTGCATCGTCCA |
| EMSA3097A | TCGCATTGCCATCATCCT |
| EMSA3260S | TGTCAAGCTACCTGCTGAACTGGCGTCGTGTTTCTGG |
| EMSA3260A | CGGGCTCAAATCGTCGTAG |
| EMSA3426S | TGTCAAGCTACCTGCTGAGGCTGCCGCTTTCCGATGCT |
| EMSA3426A | AGCGCAAAGGTTTGAGTGATTGTT |
| EMSA3456S | TGTCAAGCTACCTGCTGAGGCAGCATCAGGTCAAGG |
| EMSA3456A | GGGACAGGCTCAGCAAGG |
| EMSA3503S | TGTCAAGCTACCTGCTGAAGCGGTGGCAATCGTCG |
| EMSA3503A | GCCAGGCTCAGTTCACCAGTA |
| EMSA3557S | TGTCAAGCTACCTGCTGAGCAAAGGCACCCAGCAGT |
| EMSA3557A | GATGGAAAGTTGTCGCAAAGG |
| EMSA3613S | TGTCAAGCTACCTGCTGACCAAGGAACAACGGCACA |
| EMSA3613A | GCACGCAGTTATTCAGGGAT |
| EMSA3943S | TGTCAAGCTACCTGCTGATGTTGCAGCAATGTGGCG |
| EMSA3943A | TGCTCGAACATCTGGGTCAGT |
| EMSA4057S | TGTCAAGCTACCTGCTGATGCGTAACGCCGAAAGC |
| EMSA4057A | AGCAGCGACGTGGAAACC |
| EMSA4519S | TGTCAAGCTACCTGCTGACTCAAGGGCAAGGTCTACGC |
| EMSA4519A | CCAGGGCCAACAGTAAAGCA |
| EMSA4856S | TGTCAAGCTACCTGCTGACCGACGTGACCGATGC |
| EMSA4856A | TTGCCAGGGTCTGCTTGT |
| EMSA4888S | TGTCAAGCTACCTGCTGACAACGGCAACCAGCACC |
| EMSA4888A | GGACCGCAACGAGACCAT |
| EMSA5020S | TGTCAAGCTACCTGCTGAGAGAACCTGATCCTGGGC |
| EMSA5020A | CAGCGATTGGGCTAACC |
| EMSA5222S | TGTCAAGCTACCTGCTGACCATGTTGCGCTCGTCG |
| EMSA5222A | GCGGATTTCGTGAGGGTG |
| EMSA5270S | TGTCAAGCTACCTGCTGACCGATTACACCGCTACCAAG |
| EMSA5270A | CAGGCTGATTTCCACGAACA |
| EMSA5299S | TGTCAAGCTACCTGCTGATGGGCCTGGAGGAAGTCA |
| EMSA5299A | CGGTGTCAGTCGGCAGATAG |
| EMSA0681S | TGTCAAGCTACCTGCTGATCCACGTCTACGCGGTCAG |
| EMSA0681A | CTGCATTCGATACGGGTCA |
| EMSA0788S | TGTCAAGCTACCTGCTGATTTTCACGGGTGATGATGG |
| EMSA0788A | CCTCGGTCGATGGTTGC |
| EMSA1691S | TGTCAAGCTACCTGCTGAGGGCAACGATAAGGCACTT |
| EMSA1691A | GCGGCTCACCTGAACAAAC |
| EMSA2059S | TGTCAAGCTACCTGCTGACCTTGCCAGCCAGGTCTT |
| EMSA2059A | AGCCTTCAGCCGTTATGC |
| EMSA2858S | TGTCAAGCTACCTGCTGATGGCTGAGGCGGCACAA |
| EMSA2858A | CCACCAGTAATAGGTGATAGAGGC |
| EMSA3542S | TGTCAAGCTACCTGCTGACCGACTGAGTGCCTGTTCA |
| EMSA3542A | CAGCGGCGGGTGGTTT |
| EMSA3770S | TGTCAAGCTACCTGCTGATTTATGAACCGTCTCATTCCC |
| EMSA3770A | AACGGCGCTACCGACAA |
| EMSA3795S | TGTCAAGCTACCTGCTGACTGTTCACGCTCGTTTGCT |
| EMSA3795A | AGTGGTAGTCTTGCTGCTTGTC |
| EMSA3855S | TGTCAAGCTACCTGCTGATCGTAGGCGTTACTGAGGTT |
| EMSA3855A | AGGCCGATGCGATTCTT |
| EMSA3856S | TGTCAAGCTACCTGCTGACCGTTGACATAGACATGCTGC |
| EMSA3856A | TCCTGCGGTGGACGAAC |
| EMSA3874S | TGTCAAGCTACCTGCTGACTGAGGCTCGTTCTTGTTGA |
| EMSA3874A | CAGGCTCGTGAAGGATGG |
| EMSA4406S | TGTCAAGCTACCTGCTGACAGGTAAATACCGAGTTGGC |
| EMSA4406A | CTGTCTGCTCATCGCTGGT |
| EMSA4858S | TGTCAAGCTACCTGCTGATCGCCAGCACGGTAAGC |
| EMSA4858A | GAAGGGACAGGGTCAGGGT |
| EMSA5496S | TGTCAAGCTACCTGCTGAGGTGGCTTGCACCTGTCG |
| EMSA5496A | CCGATGCGGAAATGGC |
| EMSA5549S | TGTCAAGCTACCTGCTGAGCACAGCGGGAACGATAA |
| EMSA5549A | TCAATGGCATCTTCAACTGG |
| EMSA5560S | TGTCAAGCTACCTGCTGATCTTGCCGCCGGATTG |
| EMSA5560A | CCGATGATGACCGAGTTTGG |
| EMSA5586S | TGTCAAGCTACCTGCTGAGAACACCAGGGCGAAGAGT |
| EMSA5586A | TAGCATCACCAGCAAGAACAT |
| EMSA5592S | TGTCAAGCTACCTGCTGAACGAAATCATCGGAAGTCGC |
| EMSA5592A | TGTTCTCGGCATTGTTATAGGC |
| EMSA5710S | TGTCAAGCTACCTGCTGACAGCGGAAAGCAACCAG |
| EMSA5710A | TTGTGCATGTCCCACCC |
| FAM-tail | TGTCAAGCTACCTGCTGA |
| cyaApF1S | CCATGTTGCGCTCGTCG |
| cyaApF1A | GCCAAGGATCTGATCGTCAA |
| cyaApF2S | TGGGCACCACGATCAGG |
| cyaApF2A | GGGGTCGGATAAGCGGTA |
| cyaApF3S | GCCCATCATGCCTGAAGC |
| cyaApF3A | CCCTGGTTCAGATGCAGGAA |

^a^The restriction site regions of the primers are indicated by underline.
